# *BnaMPK3* Is a Key Regulator of Defense Responses to the Devastating Plant Pathogen *Sclerotinia sclerotiorum* in Oilseed Rape

**DOI:** 10.3389/fpls.2019.00091

**Published:** 2019-02-08

**Authors:** Zheng Wang, Ling-Li Bao, Feng-Yun Zhao, Min-Qiang Tang, Ting Chen, Yaoming Li, Bing-Xu Wang, Benzhong Fu, Hedi Fang, Guan-Ying Li, Jun Cao, Li-Na Ding, Ke-Ming Zhu, Sheng-Yi Liu, Xiao-Li Tan

**Affiliations:** ^1^Institute of Life Sciences, Jiangsu University, Zhenjiang, China; ^2^Oil Crops Research Institute, Chinese Academy of Agricultural Sciences, Wuhan, China; ^3^School of Agricultural Equipment Engineering, Institute of Agricultural Engineering, Jiangsu University, Zhenjiang, China; ^4^Faculty of Science, Jiangsu University, Zhenjiang, China; ^5^College of Life Science and Technology, Hubei Engineering University, Xiaogan, China

**Keywords:** *Brassica napus*, *Sclerotinia sclerotiorum*, *MPK3*, gain-of-function, loss-of-function, association analysis

## Abstract

The disease caused by *Sclerotinia sclerotiorum* has traditionally been difficult to control, resulting in tremendous economic losses in oilseed rape (*Brassica napus*). Identification of important genes in the defense responses is critical for molecular breeding, an important strategy for controlling the disease. Here, we report that a *B. napus* mitogen-activated protein kinase gene, *BnaMPK3*, plays an important role in the defense against *S. sclerotiorum* in oilseed rape. *BnaMPK3* is highly expressed in the stems, flowers and leaves, and its product is localized in the nucleus. Furthermore, *BnaMPK3* is highly responsive to infection by *S. sclerotiorum* and treatment with jasmonic acid (JA) or the biosynthesis precursor of ethylene (ET), but not to treatment with salicylic acid (SA) or abscisic acid. Moreover, overexpression (OE) of *BnaMPK3* in *B. napus* and *Nicotiana benthamiana* results in significantly enhanced resistance to *S. sclerotiorum*, whereas resistance is diminished in RNAi transgenic plants. After *S. sclerotiorum* infection, defense responses associated with ET, JA, and SA signaling are intensified in the *BnaMPK3*-OE plants but weakened in the *BnaMPK3*-RNAi plants when compared to those in the wild type plants; by contrast the level of both H_2_O_2_ accumulation and cell death exhibits a reverse pattern. The candidate gene association analyses show that the BnaMPK3-encoding *BnaA06g18440D* locus is a cause of variation in the resistance to *S. sclerotiorum* in natural *B. napus* population. These results suggest that *BnaMPK3* is a key regulator of multiple defense responses to *S. sclerotiorum*, which may guide the resistance improvement of oilseed rape and related economic crops.

## Introduction

Oilseed rape (*Brassica napus* L.) is an agriculturally important oilseed crop that is cultivated worldwide, including North America, Europe, and South Asia. A major constraint to its productivity is sclerotinia disease caused by the pathogen *Sclerotinia sclerotiorum* (Lib.) de Bary, resulting in a tremendous loss in seed yield: for example, 10–20% of yield losses in an average year, and up to 80% in severely infected fields in China (Oil Crop Research Institute, 1975; Wu et al., 2013). Indeed, *S. sclerotiorum* is a hugely destructive necrotrophic fungal plant pathogen that is capable of causing disease on at least 408 described plant species, including many economically important crops, and more than 60 names have been used to refer to diseases caused by this fungal pathogen in agriculture (Bolton et al., 2006). This necrotrophic pathogen exhibits little host specificity, and research on the molecular aspects underlying the interactions that it established with host plants have been mainly concentrated on fungal pathogenesis, which has revealed several virulence mechanisms of this pathogen, including secretion of numerous cell wall degrading enzymes, production of the non-host-selective toxin oxalic acid and secretion of effector proteins during infection (Riou et al., 1991, 1992; Cessna et al., 2000; Rollins and Dickman, 2001; Kim et al., 2008; Williams et al., 2011; Kabbage et al., 2013; Zhu et al., 2013; Guyon et al., 2014; Wang et al., 2015, 2018). However, the host defense to *S. sclerotiorum* in the interaction, especially in the *B. napus*-*S. sclerotiorum* interaction, is less well understood.

As the first attempts to investigate the host defense to *S. sclerotiorum*, studies on global profiles of host gene expression in response to the pathogen indicated that genes associated with jasmonic acid (JA) and ethylene (ET) signal are induced, but no salicylic acid (SA) responsive genes are identified (Zhao et al., 2007, 2009). Several latter studies showed that SA signaling, together with JA and ET signaling contribute to basal resistance against *S. sclerotiorum* (Guo and Stotz, 2007; Wang et al., 2012; Novakova et al., 2014). However, another study suggested that the resistance to the pathogen is not dependent on SA, and that JA signaling does not seem to play a predominant role in the resistance (Perchepied et al., 2010). Conclusions in these studies about the role of these signaling in resistance to *S. sclerotiorum* are contradictory. Nevertheless, these data imply that the defense responses to the pathogen involve a multiple and complicated arrange of signaling pathways.

It has been shown that mitogen-activated protein kinases (MAPKs or MPKs) play an important role in signal transduction in response to hormones and environmental stresses (Tena et al., 2001; Zhang and Klessig, 2001; Group, 2002; Nakagami et al., 2005; Meng and Zhang, 2013). An MAPK activity is controlled by sequential activation of two protein kinases, by which an MAPK kinase kinase activates an MAPK kinase that in turn activates an MAPK (Rodriguez et al., 2010). Evidence is now accumulating that some MAPK family members have been implicated in plant defense as a component of defense signaling pathways. For example, in the model plant *Arabidopsis thaliana*, microbe-associated molecular patterns (MAMPs), such as the bacterial flagellum-derived flg22 peptide, trigger the activation of AtMAPKs including AtMPK1, AtMPK3, AtMPK4, AtMPK6, AtMPK11, and AtMPK13 (Meng and Zhang, 2013; Nitta et al., 2014). Of these MAPKs, AtMPK4 was first described as a negative regulator of plant immunity (Petersen et al., 2000; Brodersen et al., 2006), whereas some others, such as AtMPK3, were believed to play positive roles in plant immunity based on their rapid activation by flg22 and pathogen inoculation (Zhang and Klessig, 2001). Further, the *Arabidopsis* thaliana *mpk3* loss-of-function mutants showed significantly lower bacterial titers of *Pseudomonas syringae*, and in expression of genes involved in SA biosynthesis and signaling, either control or flg22-treated *Atmpk3* loss-of-function mutants have no different from *Atmpk4* mutants that display enhanced resistance to biotrophic pathogens *Hyaloperonospora arabidopsidis* and *P. syringae* (Petersen et al., 2000; Frei dit Frey et al., 2014). However, another trial revealed no clear susceptible phenotype in the *Atmpk3* mutants when spray- or infiltration-inoculated with *P. syringae* (Su et al., 2017).

MPK3 genes have been identified in various crop species and implicated in defense responses and other physiological processes. In tobacco, WIPK (wound-induced protein kinase), the MPK3 ortholog in *Nicotiana tabacum*, was found to be activated during infection with TMV (tobacco mosaic virus; Zhang and Klessig, 1998a) and treatments such as SA, ET, and jasmonate in suspension cells (Zhang and Klessig, 1997, 1998b; Kumar and Klessig, 2000; Zhang et al., 2000). In *Catharanthus roseus*, MeJA application resulted in the transcript accumulation of CrMPK3 (Raina et al., 2012), suggesting that the MAPK might be involved in JA-mediated defense response. In cotton, *Gossypium barbadense MPK3* (*GbMPK3*) can be induced during multiple abiotic stress treatments including salt, cold, heat, dehydration and oxidative stress. In rice, *OsMPK3* positively regulates the JA signaling pathway and plant resistance to a chewing herbivore *Chilo suppressalis* (Wang et al., 2013). In *B. napus*, however, there have been no reports to this date on the biological functions of MPK3 in the defense of *B. napus* against *S. sclerotiorum*, one of the most important pathogens of this crop.

*B. napus* originated from a spontaneous hybridization of *Brassica rapa* L. (syn. *campestris*; AA, 2*n* = 20) and *Brassica oleracea* L. (CC, 2*n* = 18). The genome of the crop is more complex than that of the model plants *A. thaliana* or *Oryza sativa*. For example, in many cases, a gene has multiple homologous copies distributed in different loci of the *B. napus* genomic DNA, and among these homologous loci there might be a divergence of roles in contributions to their corresponding phenotypes. Candidate gene association analysis, based on numerous single nucleotide polymorphisms (SNPs) that varied across the natural population, is used to dissect the site/locus contributions to the target traits and further to validate gene function. This method was successfully applied to validate the role of genes Hd1, Hd3a and Ehd1 in controlling flowering time in cultivated rice, and showed that Hd1 proteins and Hd3a promoters as well asEhd1 expression levels were major factors affecting rice flowering (Takahashi et al., 2009). Recently, association analysis of a gene locus demonstrated that the *B. napus* DA1 locus contributed to the seed weight (Wang et al., 2017).

Diseases caused by *S. sclerotiorum* have traditionally been difficult to control (Bolton et al., 2006). Currently, breeding *S. sclerotiorum*-resistant oilseed rape cultivars using traditional methods is difficult since no immune or highly resistant germplasm in *B. napus* is available (Liu et al., 2005). Molecular breeding is pursued as an important strategy for controlling sclerotinia diseases. In this study, we used both gain- and loss-of-function approaches to investigate the specific role of *B. napus MPK3 (BnaMPK3*) in defense responses against *S. sclerotiorum*, and a candidate gene association analysis was used to validate the contribution of the genomic loci of the gene to resistance to the pathogen in a natural *B. napus* population. Our data may guide the resistance improvement of oilseed rape and related economic crops.

## Materials and Methods

### Plant and Fungal Materials

The *B. napus* cultivar Zhongshuang9 was used in this study. Plants were grown in a plant growth room under the following growth conditions: 20 ± 2°C with a photoperiod of 16 h light and 8 h dark at a light intensity of 44 μmol/m^2^/s and 60–90% relative humidity. Fresh sclerotia of the fungus *S. sclerotiorum*, collected from oilseed rape stems in the field in Zhenjiang, China, were germinated to produce hyphal inoculum on potato dextrose agar (PDA).

### Isolation of *BnaMPK3* cDNA

Total RNA from *B. napus* leaf tissues was extracted using TRIzol reagent (Invitrogen). After removal of genomic DNA contamination by DNase I (TaKaRa), 200 ng of poly(A)^+^ mRNA was converted into cDNA by MMLV Reverse Transcriptase (Promega). The cDNA template was used for PCR analysis subsequently. According to the complete CDS of *Brassica napus* cultivar Huyou15 MAPK 3 mRNA (GenBank: AY642433.1), the *BnaMPK3* cDNA was obtained using the primers *BnaMPK3*-F1 (5′-TCTTCTCATTTCAGTCCCTCA-3′) and *BnaMPK3*-R1 (5′-GGATATTTAGCCATTCATTCG-3′), cloned into PMD18-T vector (Invitrogen) according to the manufacturer’s instructions and then sequenced. The resulting plasmid pMD18-*BnaMPK3* was used as template for all experiments described below. Subcellular localization of the deduced polypeptides was predicted by Plant-mPLoc^1^ (Chou and Shen, 2010). Multiple-aligned sequences were determined by MeGa5, and GENEDOC was used to manually edit the results.

### Plasmid Construction for Transgenic Plant Generation

To construct a vector for the constitutive expression of *BnaMPK3*, the vector pCAMBIA1300-35S-Nos was generated by inserting two fragments from the vector pEGAD containing the CaMV 35S promoter and CaMV Nos terminator into the *Eco*RI/*Kpn*I and *Bam*HI/*Hin*dIII sites of pCAMBIA1300 (Dingguo Changsheng Biotech Co. Ltd., Beijing, China), respectively. The PCR primers were 5′-gaattcTTAATTAAGAGCTCGCATGCC-3′ and 5′-ggtaccGTCCCCGTGTTCTCTCCAA-3′ for the insertion of the CaMV 35S promoter. The primers were 5′-ggatccGAATTTCCCCGATCGTTCAA-3′ and 5′-aagcttGATCTAGTAACATAGATGACACCGC-3′ for the insertion of the CaMV Nos terminator. pCAMBIA1300-35S-Nos contains a hygromycin-resistant gene in its T-DNA region for selection of transgenic plants by hygromycin. Then, a 1,299 kb full-length *BnaMPK3* cDNA was PCR amplified from its cDNA clone with the primers *BnaMPK3*-F2 (5′-ggtaccTCTTCTCATTTCAGTCCCTCA-3′), *BnaMPK3*-R2 (5′-ggatccGGATATTTAGCCATT CATTCG-3′), and inserted into the *Kpn*I/*Bam*HI sites of pCAMBIA1300-35S-Nos, creating a *BnaMPK3*-overexpressing vector 1300-35S-*BnaMPK3*-NOS. The inserted sequences were confirmed by restriction enzyme digestion and sequencing, and the 1300-35S-*BnaMPK3*-NOS vector was transformed into *Agrobacterium tumefaciens* (LBA4404). For construction of RNAi vector, a fragment was amplified with a pair of primers *BnaMPK3*-F3 (5′-ACCAGGGCTTGTCTGAGGA-3′), and *BnaMPK3*-R3 (5′-AATCGCAGTTGGCGTTCA-3′), and inserted into the PMD18-T vector. The target *BnaMPK3-*RNAi fragment was cut out from the vector and then cloned into the pCXSN vector by *Kpn*I/*Bam*HI and then sequenced [Sangon Biotech (Shanghai) Co., Ltd.].

### Subcellular Localization of BnaMPK3

The *BnaMPK3* was cloned into the vector pK7FWG2.0 to create a C-terminal GFP fusion (Invitrogen, China). The nuclear marker gene *INDEHISCENT* (*IND*) was cloned into pCX-DR (Invitrogen, China) to create a C-terminal RFP fusion. These constructs were introduced into *Agrobacterium tumefaciens* (LBA4404). In these constructs, these genes were constitutively expressed under the control of the CaMV 35S promoter.

*Agrobacterium* infiltration into *Nicotiana benthamiana* leaves was performed as described previously (Wood et al., 2009). Cells of *A. tumefaciens* strain were cultured at 28°C for 2 days on Luria–Bertani (LB) agar medium containing 50 μg/mL kanamycin and 2.5 μg/mL tetracycline. The recombinant agrobacteria were grown in 10 mL LB liquid medium supplemented with appropriate antibiotics at 28°C, and then harvested by centrifugation. The cell pellet was resuspended in buffer [10 mM 2-(*N*-morpholino) ethanesulphonic acid (MES), pH 5.6, 10 mM MgCl2 and 150 μM acetosyringone], adjusted to a final optical density at 600 nm (OD600) of 0.6, and incubated for 3 h at room temperature before inoculation. *N. benthamiana* leaves were hand infiltrated using a needleless 1-mL syringe with the transformed *Agrobacterium* mixed with *Agrobacterium* containing the gene-silencing suppressor p19 (Voinnet et al., 2003). Inoculated plants were incubated at 26°C in a growth chamber for 1–2 days. Five days after transformation, fluorescence was monitored by confocal microscopy. Fluorescence emission was at 510–540 nm for GFP and 580–584 nm for RFP, and excitation was at 488 nm for GFP and 543 nm for RFP (Wood et al., 2009; Wang et al., 2014b).

For the transient expression in *N. benthamiana*, the transformation was performed as described in the relevant section of above assay of subcellular localization of *BnaMPK3*. For the transformation in *B. napus*, the plants were grown in a protected field in Zhenjiang, China, and transformed by *in planta Agrobacterium*-mediated transformation according to the procedure described by Wang et al. (2009). The transformants were examined as described in the “Results” section.

### Plant Inoculation and Chemical Treatments

Plant inoculation with *S. sclerotiorum* was performed as described previously (Dong et al., 2008; Wang et al., 2009). The experiment was in a randomized complete block design and was repeated three times. For the transgenic plants, the experiment was performed using three independent *BnaMPK3*-overexpressing or -RNAi transgenic lines, respectively. Six hours after inoculation and at intervals thereafter, the lesion size was determined as the area of the lesion after *S. sclerotiorum* infection. Chemical treatments with SA, MeJA or ABA were performed as described previously (Wang et al., 2014a,b). The leaves were sprayed with SA (100 μM), MeJA (100 μM), ABA (50 μM) or ACC (100 μM) solutions, respectively, on their leaves for 0, 3, 6, 9, or 12 h in the dark.

### Quantitative Real-Time PCR (qRT-PCR)

Total RNA was isolated from treated and control leaf samples and cDNA was synthesized as described by Wang et al. (2014a). Quantitative PCR was performed using SYBR green real-time PCR master mix in an ABI 7300 Real-Time PCR System with three technical replicates for each gene using different cDNAs synthesized from three biological replicates. *BnTIP41* (TIP41-like protein gene) was used as reference gene (Wang et al., 2014a). The relative expression levels were estimated using the 2^-ΔΔCT^ method (Livak and Schmittgen, 2001). Primers used for qPCR are listed in Supplementary Table S1. Amplification efficiencies of the primers and variation of Δ*C*T (Ct*_Target_* – Ct*_TIP41_*) with template dilution were examined and indicated that the amplification efficiencies of the target and reference are approximately equal (Supplementary File S1). These primer sets were tested by dissociation curve analysis and verified for the absence of non-specific amplification (Supplementary File S2).

### DAB Staining and Trypan Blue Staining

Procedures for DAB staining were performed as described previously (Wang et al., 2014b). For trypan blue staining, Inoculated leaves were soaked with ethanol/chloroform (3/1, vol/vol) for 24 h, stained with trypan blue solution for 4 h, and then incubated for 24 h in chloral hydrate.

### Association Analysis

The association panel consisted of 324 accessions of oilseed rape collected worldwide. All the accessions were grown in the field in Wuhan, Hubei province. The field trials were designed in a randomized complete block design with three replicates. All the accessions were evaluated for resistance to *S. sclerotiorum* in a naturally infested nursery. Thirty-six plants from each plot were rated with a disease index (Supplementary File S3) and for disease incidence at approximately the beginning of physiological maturity.

Genomic DNA was isolated from juvenile leaves of each self-pollinated lines. The polymorphic SNPs of two genomic homologous loci of *BnaMPK3* were genotyped by genomic DNA re-sequencing, and all the re-sequencing data were mapped on the reference genome “Darmor-bzh” (Chalhoub et al., 2014). The SNP variations of 324 lines were acquired using Genome Analysis ToolKit^2^. An association analysis was performed using general linear mode in Tassel software (Bradbury et al., 2007). The quantile–quantile plot was displayed with –log_10_ (*P*) of each SNP and expected *P*-value. The Manhattan plot was displayed using CMplot software^3^. The threshold of association analysis was set to *P* < 0.01.

### Statistical Analysis

Statistical analysis was performed using the SAS program (SAS Institute Inc., Cary, NC, United States). The data relating to lesion size were subjected to one-way analysis of variance followed by a comparison of the means according to a significant difference test at *P* < 0.05. Using ΔCt values (target – reference), pairwise comparisons relating to PCR were conducted according to Student’s *t*-test at *P* < 0.001, 0.001 < *P* < 0.01 or 0.01 < *P* < 0.05 under the assumption that variances are unequal.

## Results

### Cloning of *BnaMPK3* and Assessment of Its Subcellular Localization and Tissue-Specific Expression

**FIGURE 1:**
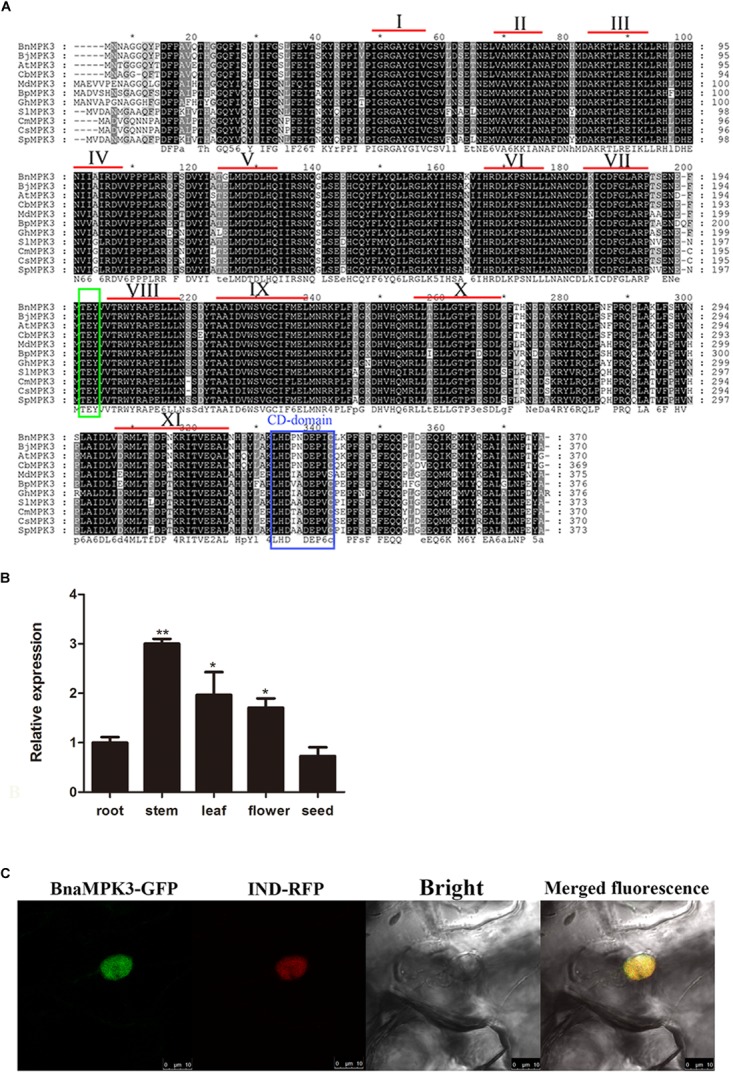
Sequence analysis of *BnaMPK3*. **(A)** Sequence alignment of BnaMPK3 and its homologs. The amino acid sequence of BnaMPK3 was aligned with those of the 10 closest matching proteins from a BLAST search. Identical amino acids are shown in black boxes, and similar amino acids are shown in gray boxes. The highly conserved domain TEY is shown in green box. The CD-domain is shown in blue box. According to Hanks and Hunter (1995), 11 subdomains (I–XI) are indicated by Roman numerals. Species abbreviations are as follows: Bn: *Brassica napus*; Bj: *Brassica juncea*; At: *Arabidopsis thaliana*; Cb: *Chorispora bungeana*; Md: *Malus domestica*; Bp: *Betula platyphylla*; Gh: *Gossypium hirsutum*; Sl: *Solanum lycopersicum*; Cm: *Cucumis melo*; Cs: *Cucumis sativus*, Sp: *Solanum peruvianum*. **(B)** Expression profiles of *BnaMPK3*. Relative expression levels of *BnaMPK3* in different tissues were determined by real-time quantitative PCR. Values are means of three replicates. The error bars show the standard deviation. The significances of between each tissue and root are indicated (Student’s *t*-test, ^∗∗∗^*P* < 0.001, ^∗∗^0.001 < *P* < 0.01 or ^∗^0.01 < *P* < 0.05). **(C)** Subcellular localization of BnaMPK3. *In planta* localization in *Nicotiana benthamiana* leaves of BnaMPK3-green fluorescent protein (GFP), IND (nuclear marker protein)-red fluorescent protein (RFP) and merged fluorescence from RFP and GFP.

A full-length cDNA was cloned from a cDNA library of *B. napus* cv. Zhongshuang9, a double low (low erucic acid-low glucosinalate) cultivar with higher resistance to the pathogen *S. sclerotiorum* than the mid-resistant cultivar Zhongyou 821 (Wang et al., 2004), by a homology cloning approach. The entire open reading frame of the cloned cDNA encodes a protein of 370 amino acid residues with a calculated molecular mass of 42.52 kDa and a predicted pI of 5.79. To identify whether the cDNA encodes the *BnaMPK3* protein, the deduced amino acid sequence was used as query in a BLASTP search of the National Center for Biotechnology Information (NCBI) database. As shown in Figure 1A, alignment of the 10 top-scoring matches with the amplified sequence indicates that sequences of all the 11 proteins from various plant species are highly similar and contain all the eleven conserved sub-domains that are characteristic of serine/threonine protein kinases (Hanks and Hunter, 1995). Similar to AtMPK3 and other plant MPK3s, the deduced sequence possess a conserved dual phosphorylation activation motif Thr-Glu-Tyr (TEY) located between sub-domains VII and VIII as well as a common docking (CD) domain in its C-terminal extension, which is considered as the typical characteristic of MAPK family and functions as a docking site for MAPKK, phosphatases, and protein substrates (Tanoue et al., 2000). Thus, according to the existing nomenclature of plant MAPKs (Group, 2002), the cloned cDNA was designated as *BnaMPK3* (GenBank accession no. KU363194).

We measured the relative expression levels of *BnaMPK3* in different plant tissues using quantitative reverse-transcription polymerase chain reaction (qRT-PCR) analysis. As shown in Figure 1B, the relative expression levels of *BnaMPK3* in roots and seeds are very similar, whereas in flowers or leaves there is almost twofold higher expression levels, whereas stems show threefold higher expression levels than roots or seeds. *S. sclerotiorum* is unusual among other necrotrophic pathogens in its requirement for flower parts that fall on to the leaves or stems. Fungal ascospores then establish in leaves, and colonize leaves and stems (Bolton et al., 2006). Thus, the tissue-specific expression profile of *BnaMPK3* may provide an expression support to the potential biological functions associated with the defense to *S. sclerotiorum*.

The subcellular localization of *BnaMPK3* was also investigated. Firstly, its amino acid sequence was analyzed in the Plant-mPLoc, a top-down strategy to augment the power for predicting plant protein subcellular localization (Chou and Shen, 2010), which predicted that BnaMPK3 has a nuclear localization. At the same time, we used TMHMM^4^ to predict *trans*-membrane helices (TMHs) of the protein, but failed to identify any TMH (data not shown). In order to examine the possible nuclear localization of BnaMPK3 *in planta*, one construct expressing *BnaMPK3* fusioned to an enhanced green fluorescent protein (eGFP) was produced. The construct, together with another construct expressing a gene fusion of the red fluorescent protein (RFP) gene and a nuclear-localized marker gene (*IND*), was transiently co-transferred by infiltration of transformed *Agrobacterium tumefaciens* cells into *N. benthamiana* leaves. As shown in Figure 1C, 5 days after infiltration, GFP and RFP fluorescence in leaf cells was associated with regions of nuclei autofluorescence, and GFP and RFP fluorescence in leave cells led to yellow fluorescence. As expected, our results indicated that BnaMPK3 is primarily located in the nucleus *in planta*.

### Expression of *BnaMPK3* in Oilseed Rape During Infection of *S. sclerotiorum* and Treatment With Hormones

**FIGURE 2:**
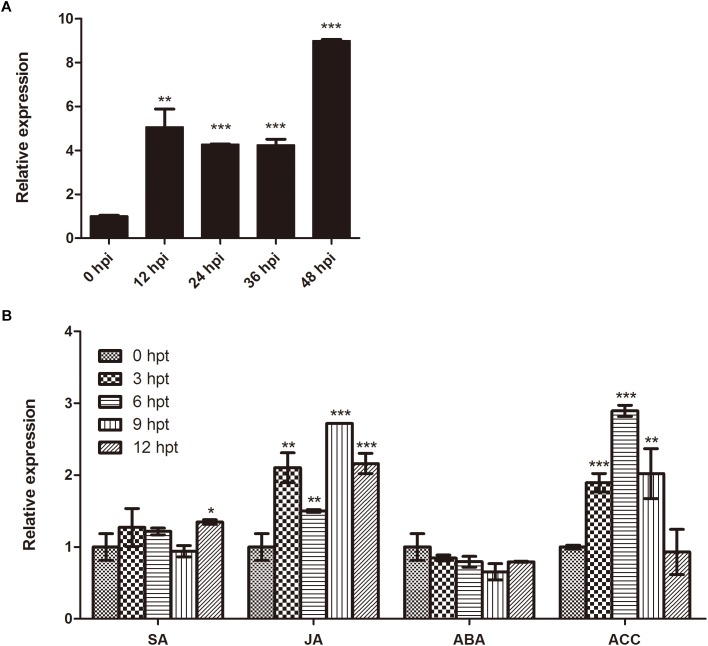
Expression of *BnaMPK3* in *B. napus* during infection of *S. sclerotiorum* and activation of plant defense responses. **(A)** Response of *BnaMPK3* to *S. sclerotiorum* infection in *B. napus*. Relative expression levels of *BnaMPK3* in *B. napus* were determined by real-time quantitative PCR at 0, 12, 24, 36, and 48 h post *S. sclerotiorum* inoculation (hpi). **(B)** Expression of *BnaMPK3* during activation of defense responses in *B. napus*. Relative expression levels of *BnaMPK3* in *B. napus* were determined by real-time quantitative PCR at 0, 3, 6, 9, and 12 h post various chemicals treated. SA, salicylic acid; MeJA, methyl jasmonate; ABA, abscisic acid; ACC, 1-aminocyclopropane-1-carboxylic acid. Values are means of three replicates. The error bars show the standard deviation. The significances of the gene expression differences between each time point and the 0-h time point are indicated (Student’s *t*-test, ^∗∗∗^*P* < 0.001, ^∗∗^0.001 < *P* < 0.01 or ^∗^0.01 < *P* < 0.05).

In order to determine whether expression of *BnaMPK3* is responsive to *S. sclerotiorum* infection, we firstly searched the published microarray data and found that the *BnaMPK3* gene was not identified in the experiment using an *Arabidopsis*-based microarray to investigate gene expression profiles in response to *S. sclerotiorum* in *B. napus*, but that an experiment using a *B. napus* oligonucleotide microarray showed that *BnaMPK3* is induced by *S. sclerotiorum* (Zhao et al., 2007, 2009). To confirm the microarray results, we examined the expression of *BnaMPK3* in *S. sclerotiorum*-infected *B. napus* plants using qRT-PCR. The results showed that the expression of *BnaMPK3* was significantly enhanced by fourfold to ninefold (*P* < 0.01 or *P* < 0.001) within 48 h post-inoculation with the fungal pathogen (Figure 2A), indicating that *BnaMPK3* is highly responsive to *S. sclerotiorum* infection in *B. napus*.

Previous studies have showed that defense to *S. sclerotiorum* in oilseed rape involved plant defense responses mediated by various plant hormones, including SA, JA, and ET as well as ABA (Wang et al., 2012; Novakova et al., 2014). Thus, we investigate the expression of the *S. sclerotiorum*-induced gene *BnaMPK3* during activation of these plant defense responses mediated by these hormones using the pharmacological approach. As shown in Figure 2B, the *BnaMPK3* gene was induced rapidly and strongly by JA and 1-aminocyclopropane-1-carboxylic acid (ACC), the natural precursor of ET biosynthesis. In contrast, the transcript expression of *BnaMPK3* was induced only slightly by SA and was even weakly restrained by ABA. The results suggested that the expression of *BnaMPK3* may be associated with the defense responses mediated by JA and ET in oilseed rape.

### *N. benthamiana* Plants Transiently Expressing *BnaMPK3* Enhance Resistance to *S. sclerotiorum*

**FIGURE 3:**
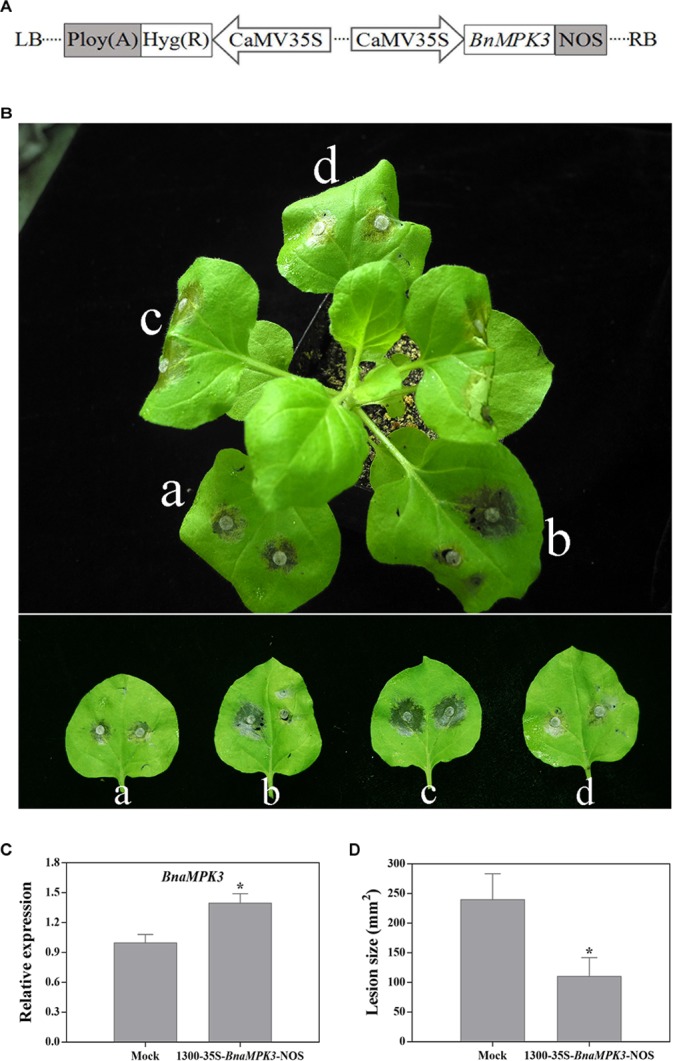
*Nicotiana benthamiana* transiently expressing *BnaMPK3* enhances resistance to *S. sclerotiorum*. **(A)** Diagram of the plasmids of 1300-35S-*BnaMPK3*-Nos used in this analysis. CaMV35S, cauliflower mosaic virus 35S promoter; NOS, terminator. **(B)** Expression of *BnaMPK3* in *N. benthamiana* enhanced resistance to *S. sclerotiorum*. (a) Both left and right panel of the leaves were treated with 1300-35S-*BnaMPK3*-Nos vector solution. (b) The left panel of a leaves was treated with mock solution, and the right panel of the leaf was treated with 1300-35S-*BnaMPK3*-Nos vector solution. (c) Both left and right panel of the leaves were treated with mock solution. (d) Both left and right panel of the leaves were treated with 1300-35S-*BnaMPK3*-Nos vector solution. **(C)** Relative expression of *BnaMPK3* in the treatment with mock or 1300-35S-*BnaMPK3*-Nos vector. **(D)** Lesion diameters were measured 36 h post-inoculation. Means and standard errors are shown. Letters indicate statistically significant differences (*P* < 0.05). The experiment was repeated three times with similar results.

*N. benthamiana* transient leaf expression system is capable of rapidly providing a clue to the gene function prior to the laborious and time-consuming stably transgenic experiment (Wood et al., 2009). Thus, to rapidly estimate the function of *BnaMPK3* in defense to *S. sclerotiorum*, we injected the leaves of *N. benthamiana* with *Agrobacterium* containing the 1300-35S-*BnaMPK3*-Nos vector that contains the cauliflower mosaic virus (CaMV) 35S promoter, *BnaMPK3* cDNA and the CaMV Nos terminator in its T-DNA (Figure 3A). Then the leaves were inoculated with *S. sclerotiorum* mycelial plugs 3-day post injection and the lesion size was determined 36 h after inoculation. To minimize the effects of variables, we also injected the left or right panel of the same leaf with *Agrobacterium* containing the 1300-35S-*BnaMPK3*-Nos vector or mock solution with *Agrobacterium* containing the 1300-35S-Nos vector, respectively, and then investigated their difference of resistance to *S. sclerotiorum*. As shown in Figure 3B–D, the 1300-35S-*BnaMPK3*-Nos-treated leaves with the higher expression level of *BnaMPK3* showed smaller lesion area than the mock-treated control, indicating that transient expression of *BnaMPK3* in *N. benthamiana* enhances resistance to *S. sclerotiorum*. These primary results in *N. benthamiana* led us to conduct further studies on oilseed rape.

### Altered Expression of *BnaMPK3* Results in Altered Resistance to *S. sclerotiorum* in *BnaMPK3*-OE and *BnaMPK3*-RNAi *B. napus* Plants

**FIGURE 4:**
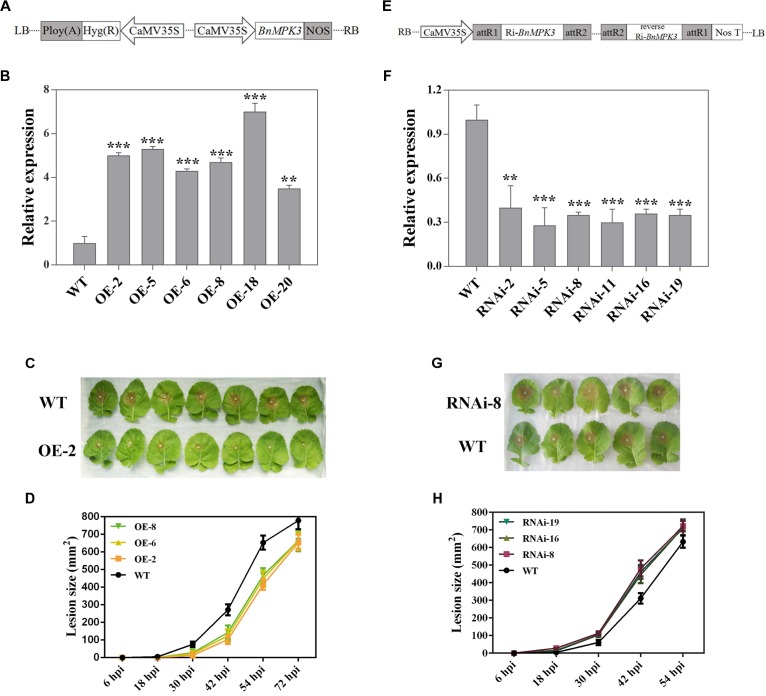
Altered expression of *BnaMPK3* results in altered resistance to *S. sclerotiorum* in oilseed rape. **(A)** Diagram of T-DNA of the plasmid used in this overexpression analysis. CaMV35S, cauliflower mosaic virus 35S promoter; NOS, terminator. **(B)** Validation of *BnaMPK3*-overexpressing lines at transcription levels revealed by real-time quantitative PCR (qRT-PCR). The significances of the gene expression differences between each *BnaMPK3*-overexpressing line and WT are indicated (Student’s *t*-test, ^∗∗∗^*P* < 0.001, ^∗∗^0.001 < *P* < 0.01 or ^∗^0.01 < *P* < 0.05). **(C)** Disease responses of inoculated plants at 42 hour post-inoculation (hpi). **(D)** Disease progression is shown from 6 to 72 hpi. Error bars indicate standard deviations. Differences in susceptibility between WT and the transgenic lines were significant (*P* < 0.05) from 18 to 54 hpi. **(E)** Diagram of T-DNA of the plasmid used in this RNAi analysis. CaMV35S, cauliflower mosaic virus 35S promoter; NOS, terminator. **(F)** Validation of *BnaMPK3*-RNAi lines at transcription levels revealed by real-time quantitative PCR (qRT-PCR). The significances of the gene expression differences between each *BnaMPK3*-RNAi line and WT are indicated (Student’s *t*-test, ^∗∗∗^*P* < 0.001, ^∗∗^0.001 < *P* < 0.01 or ^∗^0.01 < *P* < 0.05). **(G)** Disease responses of inoculated plants at 30 hpi. **(H)** Disease progression is shown from 6 to 54 hpi. Error bars indicate standard deviations. Differences in susceptibility between WT and the transgenic lines were significant (*P* < 0.05) from 18 to 42 hpi. WT, untransformed wild-type control; OE-2, -5, -6, -8, -18, -20 are six independent *BnaMPK3-*overexpressing transgenic lines; RNAi-2, -5, -8, -11, -16, -19 are six independent *BnaMPK3-*RNA-interfering transgenic lines.

In order to determine the function of *BnaMPK3* in the resistance of *B. napus* to *S. sclerotiorum*, we generated transgenic lines with overexpression (OE) of *BnaMPK3* and investigated its phenotype against *S. sclerotiorum* infection. The full-length *BnaMPK3* cDNA was cloned behind the 35S promoter and transformed into *B. napus* plants (Figure 4A). Hygromycin and PCR were used to screen *BnaMPK3* transgenic lines. Six independent transgenic lines (OE-2, -5, -6, -8, -18, and -20) showed much higher levels of expression of the *BnaMPK3* gene than the untransformed wild-type control (WT) by qRT-PCR analysis (Figure 4B), and the OE-2, -6, and -8 transgenic lines were used for further analysis.

To test the effect of *BnaMPK3* overexpression on resistance to *S. sclerotiorum*, T3 generation transgenic plants were selected by hygromycin screening and confirmed by PCR for the presence of the *BnaMPK3* transgene. Leaves from these PCR-positive plants and their untransformed controls at the six-true-leaf stage were used for inoculation with *S. sclerotiorum* mycelial plugs. The results indicated that *BnaMPK3*-OE plants developed less severe disease symptoms and showed less tissue damage than WT controls (Figure 4C). Investigation of disease progression showed that the lesion size differences between the transgenic plants and WT controls became significant (*P* < 0.05) at 18–54 h post-inoculation (hpi), and there were no significant (*P* < 0.05) differences between these transgenic lines (Figure 4D and Supplementary Figure S1), suggesting that transgenic plants did not support rapid lesion expansion when compared with WT controls. The difference in the rate of lesion expansion suggests that *BnaMPK3*-mediated defenses appear to inhibit or delay the spread of the pathogen. Ultimately, disease symptoms were limited to leaves that had been inoculated and fungal spread virtually stopped in WT controls after 72 hpi. Above all we must mention that soft-rotting necrosis occurred in WT controls as early as 18 h post-inoculation (hpi) but, in transgenic plants, the necrosis lesions are invisible in this time point, and the average lesion area of transgenic plants was reduced by ∼71, 54, and 35% at 30, 42, and 54 hpi, respectively, compared with that of WT controls (Figure 4D and Supplementary Figure S1). These results strongly suggest that overexpression of *BnaMPK3* significantly enhances oilseed rape resistance to *S. sclerotiorum* infection.

Next, we assessed the effects of the loss of *BnaMPK3* function on oilseed rape resistance to *S. sclerotiorum*. One 214-bp complementary DNA fragment and its reverse sequence were cloned into pCXSN vector under the control of the CaMV 35S promoter for subsequent generation of oilseed rape transgenic plants with the RNA interfering (RNAi)-mediated suppression of *BnaMPK3* (Figure 4E). Hygromycin and PCR were used to screen the transgenic lines. Six independent transgenic lines (RNAi-2, -5, -8, -11, -16, and -19) showed much lower expression levels of the *BnaMPK3* gene than WT controls by qRT-PCR analysis (Figure 4F), and the RNAi-8, -16, and -19 transgenic lines were used for further analysis.

To investigate the effect of decrease in the *BnaMPK3* expression on resistance to *S. sclerotiorum*, antibiotic- and PCR-positive RNAi T3 transgenic line plants were tested. As shown in Figure 4G, the *BnaMPK3*-RNAi plants developed more severe disease symptoms and showed more tissue damage than WT controls. Investigation of disease progression showed that from 18 to 42 hpi, lesion sizes of all the three transgenic lines tested were significantly larger (*P* < 0.05) than those of WT controls, and there were no significant (*P* < 0.05) differences between these transgenic lines (Figure 4H and Supplementary Figure S2), suggesting that down-expression of *BnaMPK3* enhances susceptibility to *S. sclerotiorum* infection. Ultimately, disease symptoms were limited to inoculated leaves and fungal spread virtually stopped in these RNAi plants after 54 hpi.

The BnaMPK3-OE and BnaMPK3-RNAi plants grew and developed normally, indistinguishable from WT controls, at vegetable and reproductive stages (Supplementary Figure S3). Similarly, the *Arabidopsis mpk3* loss-of-function mutants resemble wild type plants, showing normal development, and only the combination of both *mpk3* and *mpk6* mutations impairs normal development, suggesting a strongly developmental redundancy (Wang et al., 2007).

### Altered Expression of *BnaMPK3* Affects *S. sclerotiorum*-Induced Defense Responses in *BnaMPK3*-OE and *BnaMPK3*-RNAi *B. napus* Plants

**FIGURE 5:**
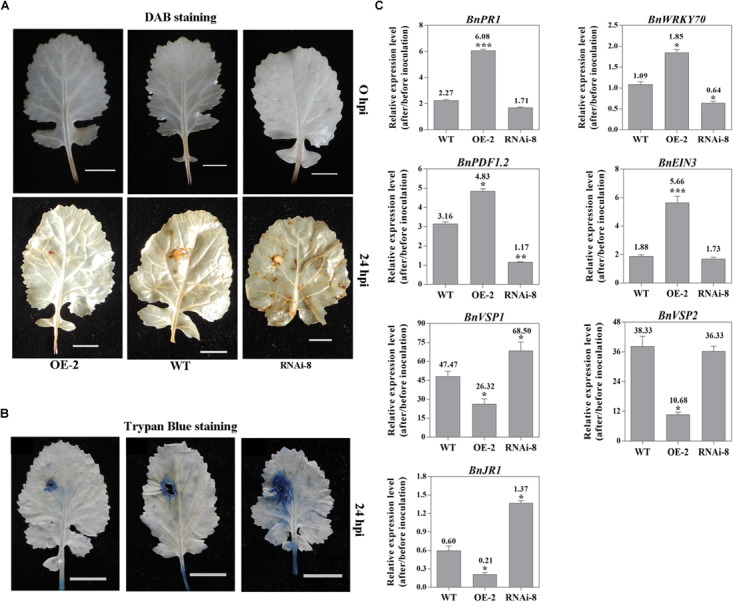
Altered expression of *BnaMPK3* affects *S. sclerotiorum*-induced defense response in *BnaMPK3*-OE and *BnaMPK3*-RNAi plants. **(A)** H_2_O_2_ accumulation in inoculated leaves. *In situ* detection of H_2_O_2_ was performed using 3,3-diaminobenzidine staining in the *BnaMPK3-*overexpressing transgenic line 2 (OE-2), the untransformed wild-type (WT) control and the *BnaMPK3-*RNA-interfering transgenic line 8 at 0 and 24 h post-inoculation with *S. sclerotiorum* (hpi). **(B)** Plant cell death was examined by using trypan blue staining on leaves of the line OE-2, WT control and the line RNAi-8. **(C)** Changes in expression of defense genes associated with SA, JA, and ET defense signaling in OE-2, WT, and RNAi-8 plants after 24 hpi. Samples were collected at the indicated time for total RNAs isolation. Expressions of these genes were quantified by real-time PCR, and then change of gene expression (folds of change relative to the level before inoculation) was calculated. Values are means of three replicates, and error bars indicate standard deviations. The significances of the gene expression differences between each transgenic line and WT are indicated (Student’s *t*-test, ^∗∗∗^*P* < 0.001, ^∗∗^0.001 < *P* < 0.01 or ^∗^0.01 < *P* < 0.05). These above experiments were repeated with the *BnaMPK3-*overexpressing lines 6 and 8, and the *BnaMPK3*-RNAi lines 16 and 19, respectively, and results are similar.

To examine whether altered expression of *BnaMPK3* affected the defense responses under the infection by *S. sclerotiorum*, we analyzed and compared the patterns for accumulation of reactive oxygen species (ROS) and expression of defense genes after the pathogen infection in the WT as well as *BnaMPK3*-OE and *BnaMPK3*-RNAi lines. In assays for *in situ* detection of ROS, accumulation of H_2_O_2_, a major component of ROS, in leaves was detected by 3,3-diaminobenzidine (DAB) staining. As shown in Figure 5A, without pathogen challenge, no difference in accumulation of H_2_O_2_ was seen among different plant lines. In turn, at 24 h after inoculation by *S. sclerotiorum*, accumulation of H_2_O_2_ remarkably increased in inoculated leaves. In turn, more staining was observed in leaves of *BnaMPK3*-RNAi plants exhibiting enhanced susceptibility to *S. sclerotiorum* while less in leaves of *BnaMPK3*-OE plants exhibiting enhanced resistance to the pathogen, as compared to that in WT controls (Figure 5A). The ROS accumulation is one of most universal responses observed in plants following pathogen challenge (Apel and Hirt, 2004), and accumulating evidence has shown that the induction of plant ROS by *S. sclerotiorum* infection leads to cell death in host tissue and that the control of cell death governs the outcome of the *S. sclerotiorum*–plant interaction (Dickman et al., 2001; Kim et al., 2008; Williams et al., 2011; Kabbage et al., 2013). Thus, we investigated the cell death extent using Trypan Blue staining when H_2_O_2_ accumulates in the infected leaves. As shown in Figure 5B, in response to *S. sclerotiorum* infection, plant cell death as shown by trypan blue staining is in agreement with the accumulation of H_2_O_2_ as shown by DAB staining. These results demonstrate that the resistance-enhanced *BnaMPK3*-overexpressing plants inhibited both H_2_O_2_ accumulation and cell death in response to *S. sclerotiorum* infection, whereas the susceptibility-enhanced RNAi plants intensify them, when compared with WT controls.

On the other hand, we investigate changes in the expression of defense genes associated with SA, JA, and ET defense signaling in the three kinds of plants after *S. sclerotiorum* infection. Seven signaling marker genes were selected to represent the well-characterized SA- and JA/ET-dependent defense pathways, including SA signaling marker genes *PR1*, encoding a pathogenesis-related protein1, and *WRKY70*, encoding a plant-specific transcription factor WRKY70 that is downstream of NPR1 in SA signal pathway, the JA/ET signaling marker gene *PDF1.2*, encoding a JA/ET-responsive plant defensin, JA responsive genes including VEGATATIVE STORAGE PROTEIN1 (*VSP1*), *VSP2* and JASMONATE RESPONSIVE1 (*JR1*), and the ET signaling marker gene *EIN3*, encoding the critical transcription factor that is downstream of EIN2, which quickly accumulate in the presence of ET and confer specificity to ET response (Solano et al., 1998; Rojo et al., 1999; Guo and Ecker, 2003; Potuschak et al., 2003; Gagne et al., 2004; Li et al., 2004; Lorenzo et al., 2004; An et al., 2010; Zhao and Guo, 2011; Ji and Guo, 2013). The qRT-PCR analyses indicated that all the signaling marker genes exhibited enhanced expression in WT controls after *S. sclerotiorum* infection, which is in good agreement with the previous observation (Wang et al., 2012; Novakova et al., 2014). However, the expression of *BnPR1, BnWRKY70, BnPDF1.2* and *BnEIN3* was higher in the plants overexpressing *BnaMPK3* and was lower in the plants down-expressing *BnaMPK3* than in WT controls, whereas *BnVSP1, BnVSP2* and *JR1* exhibit inverse expression pattern by comparison (Figure 5C). The results suggest that altered expression of *BnaMPK3* affects the pathogen-induced defense responses including ROS accumulation, cell death and expression of defense genes associated with SA, JA, and ET defense signaling in *BnaMPK3*-OE and *BnaMPK3*-RNAi plants, and the expression of these defense responses is generally in an inverse pattern between the *BnaMPK3*-OE and the *BnaMPK3*-RNAi plants.

### *BnaMPK3* Differentially Regulates the Expression of SA, JA, and ET Biosynthesis Genes in Response to *S. sclerotiorum* Infection

**FIGURE 6:**
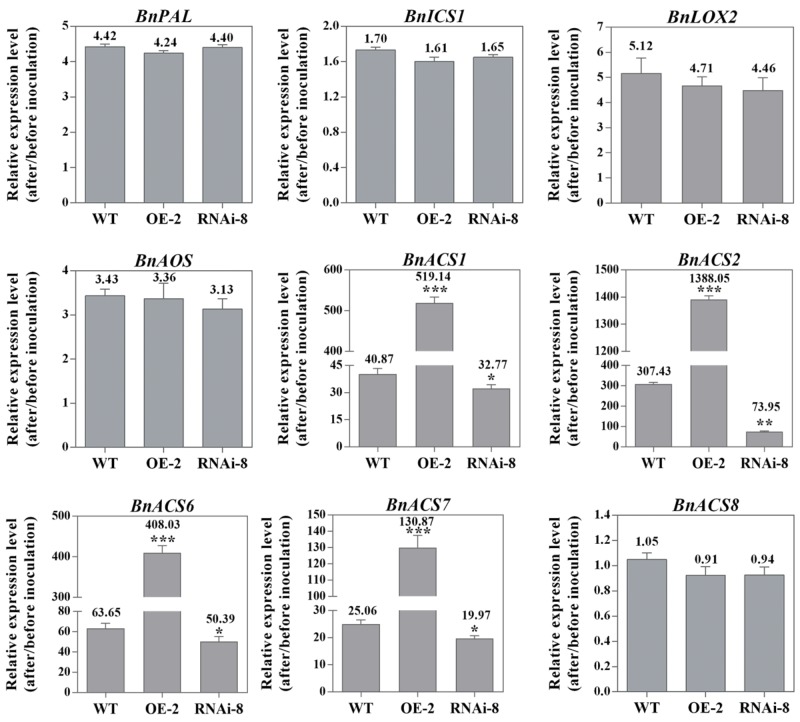
*BnaMPK3* differentially regulates the expression of SA, JA, and ET biosynthesis genes in response to *S. sclerotiorum* infection. Changes in expression of the genes involving in SA, JA, and ET biosynthesis in the *BnaMPK3-*overexpressing transgenic line 2 (OE-2), the untransformed wild-type (WT) control and the *BnaMPK3-*RNA-interfering transgenic line 8 (RNAi-8) plants after 24 hpi. Samples were collected at the indicated time for total RNAs isolation. Expressions of these genes were quantified by real-time PCR, and then change of gene expression (folds of change relative to the level before inoculation) was calculated. Values are means of three replicates, and error bars indicate standard deviations. The significances of the gene expression differences between each transgenic line and WT are indicated (Student’s *t*-test, ^∗∗∗^*P* < 0.001, ^∗∗^0.001 < *P* < 0.01 or ^∗^0.01 < *P* < 0.05). These experiments were repeated with the *BnaMPK3-*overexpressing lines 6 and 8, and the *BnaMPK3*-RNAi lines 16 and 19, and results are similar.

We have shown that the marker genes associated with SA, JA, and ET defense signaling are differentially express in different plant lines under *S. sclerotiorum* infection. In order to have a deeper understand of these important signaling pathways, we next tested if the biosynthetic genes of the three important defense signaling molecules SA, JA, and ET have corresponding expression profiles. For SA, we selected phenylalanine ammonia lyase gene (*PAL*) and isochorismate synthase gene *(ICS1*), two key SA biosynthesis genes (Lee et al., 1995; Wildermuth et al., 2001). The results showed that the two genes have not significant difference in transcript level among different plant lines, though they all are induced by *S. sclerotiorum* (Figure 6). Similar expression profiles appear also in lipoxygenase gene (*LOX2*) and allene oxide synthase gene (*AOS*), two key JA biosynthesis genes (Figure 6) (Sasaki et al., 2001; Park et al., 2002; von Malek et al., 2002). The results suggested that the expression of SA and JA biosynthesis genes in response to *S. sclerotiorum* infection is independent on *BnaMPK3*, though the pathogen can induce their expression.

In ET biosynthesis, the rate-limiting enzyme is ACS (1-amino-cyclopropane-1-carboxylic acid synthase, ACC synthase), encoded by a small gene family in plants, and based on the presence/absence of phosphorylation sites in their C-termini. The different ACS isoforms have been classified into three types (Yoshida et al., 2005). In the study, to test whether *BnaMPK3* regulates the three types ACS isoforms in the interaction between the crop oilseed rape and *S. sclerotiorum*, we selected the Type I ACS genes *BnACS1, BnACS2* and *BnACS6*, the Type II gene *BnACS8* and the Type III gene *BnACS7*. As shown in Figure 6, transcripts of *BnACS1, BnACS2, BnACS6* and *BnACS7* accumulated approximately 40, 307, 63, and 25 folds, respectively, over their basal levels in the untransformed WT controls in response to *S. sclerotiorum* infection. However, when observed on the transgenic plants, we found that their transcripts were further elevated to about 519, 1388, 408, and 130 folds, respectively, in the *BnaMPK3*-OE plants, whereas they were suppressed in *BnaMPK3*-RNAi plants exhibiting about 32, 73, 50, and 19 folds, respectively, over their basal levels. In contrast, transcript of *BnACS8* was not affected in different plant lines when infected by the pathogen. These results showed that *BnaMPK3* is a positive regulator of Type I ACS genes *BnACS1, BnACS2* and *BnACS6* and the Type III gene *BnACS7* in response to *S. sclerotiorum* infection.

### Association Analysis of the *BnaMPK3* Homologous Copies in the Genomic DNA

Through a BLAT search in *B. napus* genomic database^5^, the coding sequence of *BnaMPK3* was mapped into the genomic region of 44389463–44391236 (*BnaC03g55440D*) of the chrC03 chromosome and the other genomic region of 10591788–10593582 (*BnaA06g18440D*) of the chrA06 chromosome of *B. napus* cv. ZS11 (Chalhoub et al., 2014), indicating that the gene has two homologous copies in the genomic DNA, and sequence comparison showed that the two copies have the same exon–intron organization (Supplementary Figures S4, S5).

**FIGURE 7:**
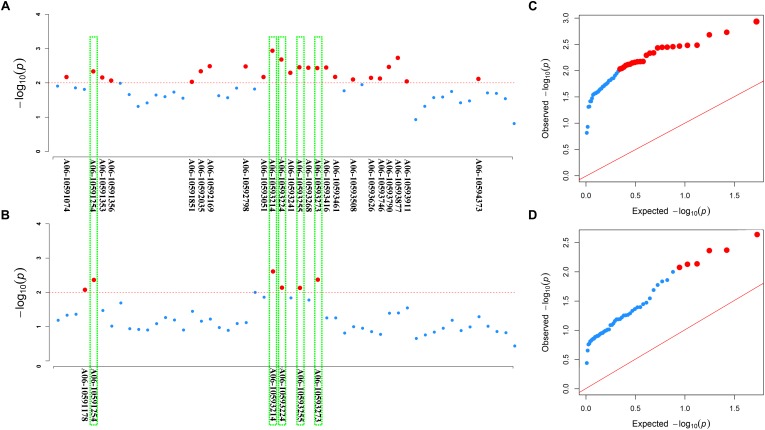
Association of SNP polymorphisms with disease index or disease incidence rate across the *BnaA06g18440D* locus of *BnaMPK3*. **(A)** Manhattan plot for disease index or **(B)** disease incidence rate. The threshold of association analysis was set to *P* < 0.01. The red-dotted line was –log_10_(*p*) = –log_10_(0.01) = 2.0, and the red dot above the red-dotted line represents a significantly associated SNP. The red dots in the green box are significantly associated SNPs for both disease index and disease incidence rate. **(C)** The Q–Q plots for disease index or (D) disease incidence rate from association analysis. The red line was the unbiased estimates of the expected and observed value. Red dots were the significant SNPs.

Furthermore, we used association analysis of a set of 324 accessions collected from different geographic position to dissect roles of the two loci *BnaC03g55440D* and *BnaA06g18440D* in contributing to the resistance to *S. sclerotiorum*. In the natural *B. napus* population, disease index varied from 7.14 to 100, and disease incidence rate varied from 19 to 100%. The results displayed there are 24 and 6 significantly associated SNPs for disease index and disease incidence rate, respectively, were detected in the locus *BnaA06g18440D* by re-sequencing (Figure 7A,B). For disease index, the 24 significantly associated SNPs explain 3.56–4.88% of the variation in this population, and for disease incidence rate, the six significantly associated SNPs explain 3.60–4.39%. And of these, five SNPs are for both disease index and disease incidence rate (Figure 7A,B). In the QQ plot, the observed value of these SNPs significantly deviates from expected value (Figure 7C,D), indicating that they were associated with the resistance to *S. sclerotiorum*. In contrast, in the locus *BnaC03g55440D*, no significantly associated SNPs for both disease index and disease incidence rate are found (data not shown), indicating that for the two homologs of *BnaMPK3, BnaA06g18440D*, but not *BnaC03g55440D*, is a cause of variation in the resistance to *S. sclerotiorum* in natural *B. napus* population.

## Discussion

In this study, we provide new data that enlarge the understanding of *MPK3* functions, indicating that *BnaMPK3*, an *MPK3* ortholog in *B. napus*, plays an important role in the defense against *S. sclerotiorum*, the most important pathogen of the crop. Our results show that not only is the expression of *BnaMPK3* highly responsive to the necrotrophic *S. sclerotiorum* infection, but it is also induced by JA and the biosynthesis precursor of ET, two signaling molecules associated with defense against necrotrophic pathogens (Wang et al., 2012). Further, overexpression of *BnaMPK3* in *B. napus* and *N. benthamiana* results in significantly enhanced resistance to *S. sclerotiorum*, the RNAi transgenic plants significantly reduce the resistance, and the expression of defense responses is generally in an inverse pattern between the *BnaMPK3*-OE and the *BnaMPK3*-RNAi plants. These results indicate a clear biological function of *BnaMPK3* in the host defense of the crop to the agriculturally devastating plant pathogen. Unlike *Arabidopsis, B. napus*, the allotetraploid species, usually has multiple homologs with putatively redundant functions on the A and C genomes. To alter a monogenic trait for evaluating the role of the target gene, therefore, it is necessary to combine mutated homologs from both subgenomes (Wells et al., 2014; Emrani et al., 2015). Indeed, in this study, the RNAi construct can down-regulate the expression of both *BnaA06g18440D* and *BnaC03g55440D*, the two homologs from A and C subgenomes, respectively, because both *BnaA06g18440D* and *BnaC03g55440D* contain the RNAi sequence (Supplementary Figures S4, S5). Further, the candidate gene association analysis yield additional insights and suggest that the allelic variation in *BnaA06g18440D*, the homeolog on A genomes, is a source of resistance diversity in cultivated *B. napus*. We also noticed that the phenotypic contribution of these significant SNPs, detected by association analysis, was low. The reason may be that the resistance to *S. sclerotiorum* is a complex quantitative trait controlled by many quantitative trait loci (QTLs) (Bert et al., 2002; Liu et al., 2005; Zhao et al., 2006). In fact, most of the QTLs identified through QTL mapping studies also show small effects on the resistance to *S. sclerotiorum* in *B. napus* (Zhao, 2003; Zhao et al., 2006; Yin et al., 2010; Wu et al., 2013; Wei et al., 2014). A similar phenomenon also is observed in the resistance of soybean to the pathogen, in which many of the SNPs explains 3.2–5.1% of the variation in the soybean population (Wen et al., 2018). Thus, these results suggested that the resistance to *S. sclerotiorum* is a trait with very complex genetic basis determined by multiple minor QTLs.

The positive role of *BnaMPK3* in the defense responses is closely associated with high expression levels of *BnACS* genes. First, the expression of *BnACSs*, consistent with *BnaMPK3*, is highly responsive to *S. sclerotiorum* infection. For example, *S. sclerotiorum*-responsive expression of *BnACS2* increased by more than 300 folds relative to its basal level. Further, the expression level of the *BnACSs* was striking elevated in the *BnaMPK3*-overexpressing resistant plants and suppressed in the *BnaMPK3*-downexpressing susceptible plants. Especially in the case of *BnACS2*, its elevated expression level is up to about 1400 folds in the *BnaMPK3*-OE plants. These results indicate that *BnaMPK3* strongly positively regulates ET signaling in response to *S. sclerotiorum* infection. This is also supported by the expression of *BnEIN3*, a key gene that regulates most, if not all, of the ET responsiveness (Alonso et al., 2003; An et al., 2010), in *BnaMPK3*-OE plants (Figure 5). Previously, studies on *Arabidopsis* showed that the mutant *eto3*, affected in the regulation of ET signaling (Chae et al., 2003), is significantly affected in resistance to *S. sclerotiorum*, and the ET-insensitive mutant *ein2-1* showed a significantly increased susceptibility to the fungus (Guo and Stotz, 2007; Perchepied et al., 2010). Thus, taking these data together, it is possible that the positive regulation of ET signaling by *BnaMPK3* plays a key role in the defense response to *S. sclerotiorum*. On the other hand, in contrast to the positive effect of *BnaMPK3* on ET signaling, we also observed that application of ACC, the biosynthesis precursor of ET, results in the activation of *BnaMPK3* expression, indicating that the expression of *BnaMPK3* and ET biosynthesis genes forms a positive feedback loop in response to *S. sclerotiorum* infection. Thus, our results suggest that the activation of ET defense signaling is important for the resistance conferred by *BnaMPK3*.

Previously, based on the microarray data, Zhao et al. (2009) detected that *S. sclerotiorum* induces the type I *BnACS6* expression in *B. napus* plants. Recently, Novakova et al. (2014) reported that in addition to *BnACS6, BnACS2*, another type I *ACS*, can also be induced in response to the pathogen infection. Here, our data suggest that in addition to *BnACS2* and *BnACS6*, the Type I ACS gene *BnACS1* and the Type III gene *BnACS7* are also highly responsive to *S. sclerotiorum* infection, and further indicate that the expression of these *BnACSs* is positively regulated by *BnaMPK3*. By contrast, in the case of the Type II *BnACS8*, while the gene cannot be induced by *S. sclerotiorum* infection, its expression is also not affected by the alteration of *BnaMPK3* expression, suggesting differential implication of these types of enzymes in response to the pathogen and in the regulatory mechanism. Additionally, we observed that the reduced magnitude of expression of these pathogen-responsive *BnACS* genes as well as other genes in the *BnaMPK3*-RNAi plants is obviously less than the elevated magnitude in *BnaMPK3*-overexpressing plants. This may be due to overlapping function shared by others in activating the expression of these genes in the interaction of *B. napus*-*S. sclerotiorum*, as observed in the *Arabidopsis* and *B. cinerea* interaction in which the expression of *AtACS2* and *AtACS6* genes decrease only slightly in the *mpk3* or *mpk6* single mutant, whereas greatly decrease in *mpk3*/*mpk6* double mutant, suggesting the absence of only one MAPK may not be sufficient to block *ACSs* activation (Li et al., 2012).

By contrast, the expression change of *BnAOS* and *BnLOX2* in *BnaMPK3*-OE or *BnaMPK3*-RNAi lines had not significant difference from that in the WT after *S. sclerotiorum* infection, suggesting that the two JA-biosynthetic genes do not appear to be involved in the defense responses mediated by *BnaMPK3*. Previously, it has been shown that *JAR1*, another JA signaling gene encoding an enzyme that generates the jasmonyl-isoleucine (JA-Ile) conjugate (Staswick and Tiryaki, 2004), does not play a role in the resistance because the jasmonate-resistant mutant *jar1-1* was not affected for responsiveness to *S. sclerotiorum* and showed a completely wild-type phenotype in response to the pathogen (Perchepied et al., 2010), though exhibiting enhanced sensitivity to another fungal necrotrophic *Pythium irregulare* (Adie et al., 2007). However, the *Arabidopsis* mutant *coi1-1* that completely blocks both JA- and ET-induced *PDF1.2* expression (Pré et al., 2008; Perchepied et al., 2010) is highly susceptible to *S. sclerotiorum* (Guo and Stotz, 2007), suggesting a role for a cooperation of JA and ET signaling. It has been well established that ET and JA signaling interact both synergistically and antagonistically in *Arabidopsis* (Glazebrook, 2005; Pieterse et al., 2009). For instance, some genes, including *EIN3* and *PDF1.2*, are synergistically activated by JA and ET (Chao et al., 1997; Solano et al., 1998; Alonso et al., 2003; An et al., 2010), whereas JA-mediated activation of other genes, including *VSP1, VSP2*, and *JR1*, are suppressed by ET (Rojo et al., 1999; Lorenzo et al., 2004). The two signaling interactions are compatible with our observation in which the expression of *BnEIN3* and *BnPDF1.2* is positively correlated with that of *BnACSs* in *BnaMPK3*-OE or –RNAi plants, whereas *BnVSP1, BnVSP2*, and *BnJR1* exhibit negative correlation, suggesting that the different expression patterns of these JA and/or ET signaling genes are outcomes of the interaction between the two signaling.

It is generally accepted that SA signaling has a central role in response to biotrophic pathogens, but mutant affected in the signaling showed significantly reduced resistance to the necrotrophic *S. sclerotiorum* in *Arabidopsis* (Guo and Stotz, 2007). In favor of a role of the signaling in this interaction between *B. napus* and *S. sclerotiorum*, is the recent discoveries that exogenous SA induces resistance to the pathogen (Li et al., 2012; Novakova et al., 2014). Further supporting the role of SA signaling in the resistance, the expression of *BnPR1* and *BnWRKY70*, two SA signaling downstream genes, are significantly elevated expression in the resistance-enhanced *BnaMPK3*-overexpressing plants, although the expression of SA biosynthesis genes *BnICS1* and *BnPAL* are not affected by *BnaMPK3*, suggesting that *BnaMPK3* act in the downstream of SA signaling to activate the defense response that takes part in the contributions to the resistance. Recently, it was suggested that there is a potential short biotrophic phase in the lifestyle of *S. sclerotiorum*, and a new model depicting the lifestyle transition of the pathogen from biotrophic to necrotrophic growth was proposed (Kabbage et al., 2015). Thus, our previous observations suggest that SA defense signaling might function on the biotrophic phase of the pathogen.

*BnaMPK3* might involve redox control to be in favor of inhibition to the *S. sclerotiorum* infection, as cell death caused by the infection is significantly inhibited in H_2_O_2_ accumulation-suppressed *BnaMPK3*-OE plants. Previously, Kim et al. (2008) have showed that when ROS induction is inhibited, programmed cell death (PCD) induced by oxalic acid, an important pathogenicity determinant of *S. sclerotiorum*, does not occur and the PCD response is required for disease development of the pathogen. Recent studies showed that the control of cell death governs the outcome of the *S. sclerotiorum*–plant interaction (Kabbage et al., 2013) and, once infection is established, the *S. sclerotiorum* induces the generation of plant ROS, leading to PCD of host tissue, the result of which is of direct benefit to the pathogen (Williams et al., 2011). These results are also supported by our observation in which the *S. sclerotiorum*-induced H_2_O_2_ accumulation is negatively correlated with the altered resistance in the OE and RNAi plants, suggesting that the control of ROS accumulation is another defense mechanism of the resistance conferred by *BnaMPK3* against *S. sclerotiorum*.

In summary, our study reveals that *BnaMPK3* is a key regulator of defense responses to *S. sclerotiorum* in oilseed rape. These data, especial those in the analysis on the overexpression of the gene, suggested that *BnaMPK3* is a promising gene for crop improvement, and that the *BnaA06g18440D* could be developed as a functional molecular marker in marker assistant breeding for the improvement in resistance to *S. sclerotiorum* because of the allelic variation of the locus in cultivated *B. napus*.

## Author Contributions

ZW and X-LT designed the experiments. ZW, L-LB, F-YZ, TC, HF, and G-YL carried out the experiments. ZW, X-LT, S-YL, YL and BF analyzed experimental results. ZW, JC, B-XW, L-ND, and K-MZ analyzed sequencing data and did statistical analysis. M-QT and B-XW analyzed GWAS data and made figures. ZW wrote the manuscript. All authors read and approved the final manuscript.

## Conflict of Interest Statement

The authors declare that the research was conducted in the absence of any commercial or financial relationships that could be construed as a potential conflict of interest.

## Footnotes

1 http://www.csbio.sjtu.edu.cn/bioinf/plant-multi/

2 https://software.broadinstitute.org/gatk/

3 https://github.com/YinLiLin/R-CMplot

4 http://www.cbs.dtu.dk/services/TMHMM-2.0/

5 http://www.genoscope.cns.fr/blat-server/cgi-bin/colza/webBlat

## Supplementary Material

FIGURE S1 Disease progression of *Sclerotinia sclerotiorum in BnaMPK3* overexpression lines and the untransformed wild-type control. The pictures were taken at leaves from seven plants of WT and seven hygromycin- and PCR-positive plants of line 2. WT means the untransformed wild-type control; OE-2 means the *BnaMPK3-*overexpressing transgenic line 2; hpi means hour post-inoculation.

FIGURE S2 Disease progression of *Sclerotinia sclerotiorum in BnaMPK3* RNAi lines and the untransformed wild-type control. The pictures were taken at leaves from five plants of WT and five hygromycin- and PCR-positive plants of line 8. WT means the untransformed wild-type control; RNAi-8 means the *BnaMPK3-*RNA-interfering transgenic line 8; hpi means hour post-inoculation.

FIGURE S3 The *BnaMPK3*-OE and *BnaMPK3*-RNAi line plants grow and develop normally at vegetable and reproductive stages. **(A)** Seedling height. **(B)** Seedling fresh weight. **(C)** Plant height in the mature stage. **(D)** Efficient primary branches. **(E)** Liliques on main inflorescence. **(F)** Efficient siliques/plant. **(G)** Seeds/silique. **(H)** 1000 seeds weight. **(I)** Whole plant seeds weight. Value represents mean and error bars indicate standard deviations from three independent rapeseed samples. There were no significant difference between transgenic lines (OE-2 or RNAi-8) and WT (*P* < 0.05). These above experiments were repeated with the *BnaMPK3-*overexpressing lines 6 and 8, and the *BnaMPK3*-RNAi lines 16 and 19, respectively, and results are similar.

FIGURE S4 Sequence alignment of *BnaMPK3* and *BnaC03g55440D*. The coding sequence of *BnaMPK3* was aligned with *BnaC03g55440D* from a BLAT search. Identical nucleotide bases are shown in black boxes. The intron sequences are shown in the yellow boxes. The RNAi sequences are showed within the red frame.

FIGURE S5 Sequence alignment of *BnaMPK3* and *BnaA06g18440D*. The coding sequence of *BnaMPK3* was aligned with *BnaA06g18440D* from a BLAT search. Identical nucleotide bases are shown in black boxes. The intron sequences are shown in the yellow boxes. The RNAi sequences are showed within the red frame.

TABLE S1 Primers used for qRT-PCR.

FILE S1 The efficiencies (E) of the primer-specific polymerase chain reaction (PCR) amplifications and variation of Δ*C*T (Ct*_Target_* - Ct*_TIP41_*) with template dilution.

FILE S2 Dissociation curves for all amplicons generated by all primer sets.

FILE S3 A disease index used to evaluate the symptom severity at maturity.
