# Supplementary figures and images for: BnaMPK3 Is a Key Regulator of Defense Responses to the Devastating Plant Pathogen Sclerotinia sclerotiorum in Oilseed Rape
Source: Front Plant Sci. 2019 Feb 8;10:91. doi: 10.3389/fpls.2019.00091 (PMC6376111; doi:10.3389/fpls.2019.00091)

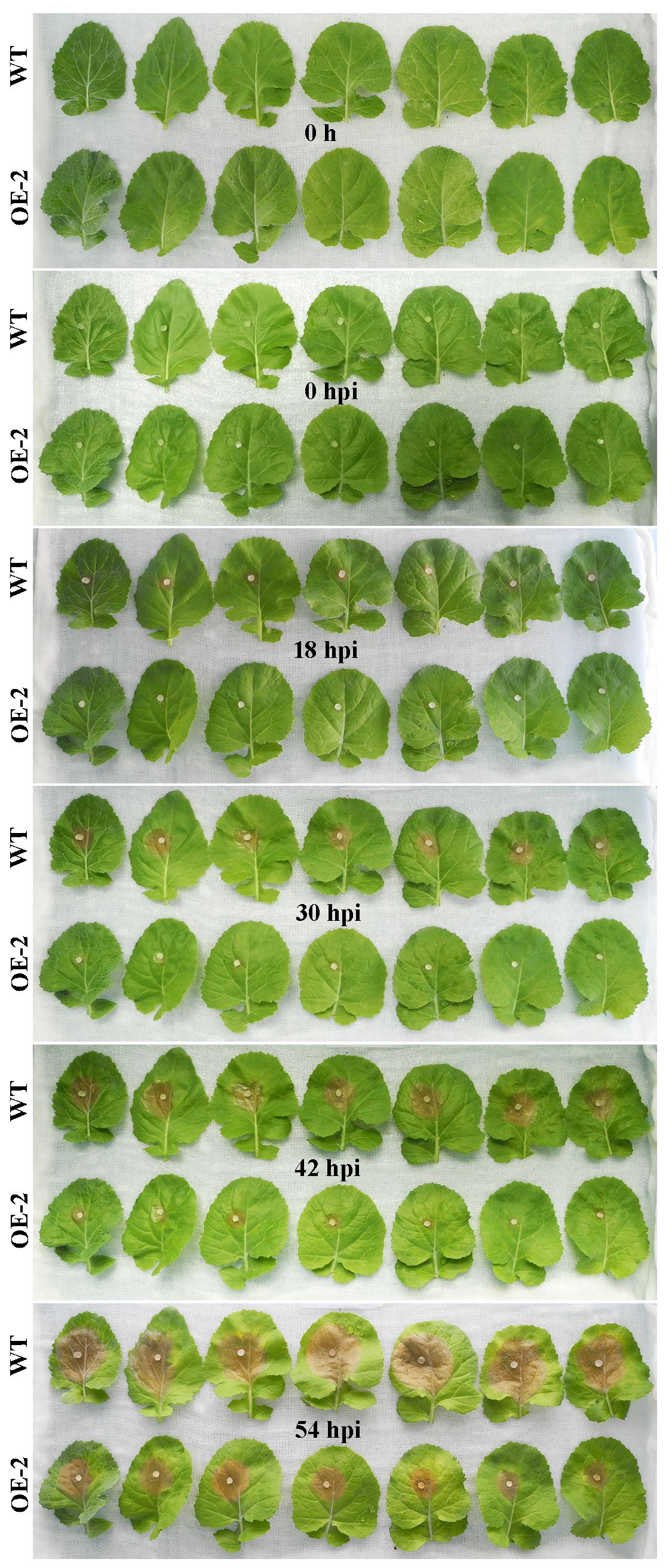

Supplement: FIGURE S1 — Disease progression of Sclerotinia sclerotiorum in BnaMPK3 overexpression lines and the untransformed wild-type control. The pictures were taken at leaves from seven plants of WT and seven hygromycin- and PCR-positive plants of line 2. WT means the untransformed wild-type control; OE-2 means the BnaMPK3-overexpressing transgenic line 2; hpi means hour post-inoculation. [file Image_1.TIF]

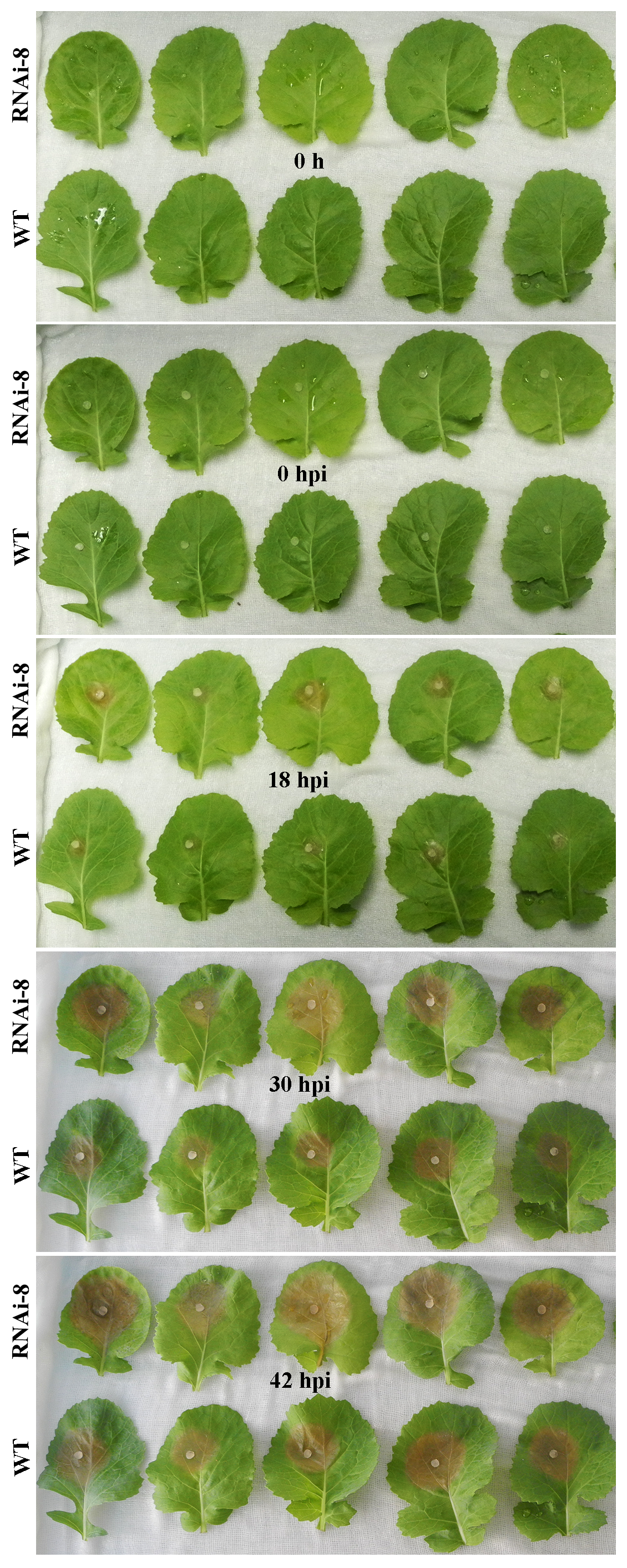

Supplement: FIGURE S2 — Disease progression of Sclerotinia sclerotiorum in BnaMPK3 RNAi lines and the untransformed wild-type control. The pictures were taken at leaves from five plants of WT and five hygromycin- and PCR-positive plants of line 8. WT means the untransformed wild-type control; RNAi-8 means the BnaMPK3-RNA-interfering transgenic line 8; hpi means hour post-inoculation. [file Image_2.TIF]

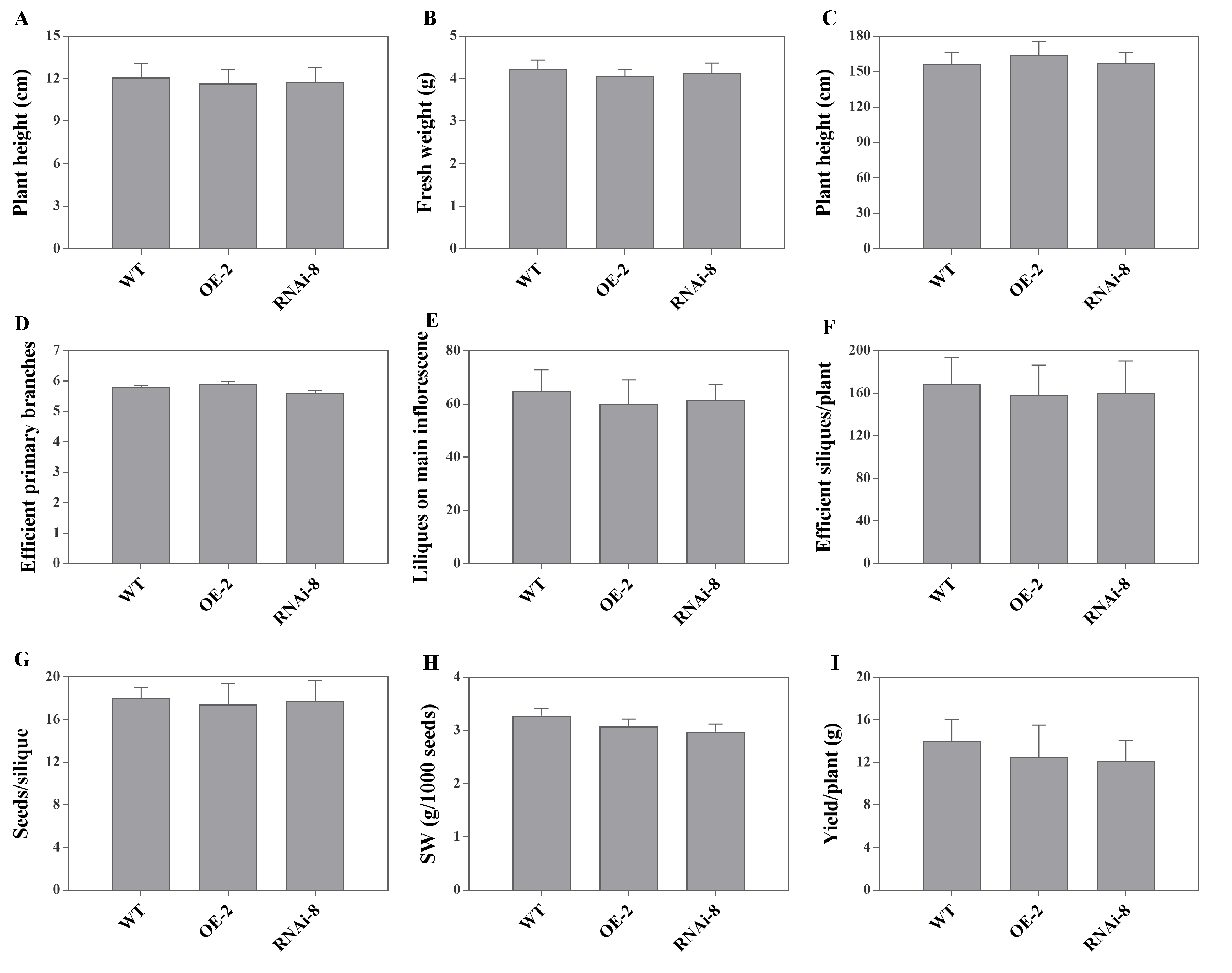

Supplement: FIGURE S3 — The BnaMPK3-OE and BnaMPK3-RNAi line plants grow and develop normally at vegetable and reproductive stages. (A) Seedling height. (B) Seedling fresh weight. (C) Plant height in the mature stage. (D) Efficient primary branches. (E) Liliques on main inflorescence. (F) Efficient siliques/plant. (G) Seeds/silique. (H) 1000 seeds weight. (I) Whole plant seeds weight. Value represents mean and error bars indicate standard deviations from three independent rapeseed samples. There were no significant difference between transgenic lines (OE-2 or RNAi-8) and WT (P < 0.05). These above experiments were repeated with the BnaMPK3-overexpressing lines 6 and 8, and the BnaMPK3-RNAi lines 16 and 19, respectively, and results are similar. [file Image_3.TIF]

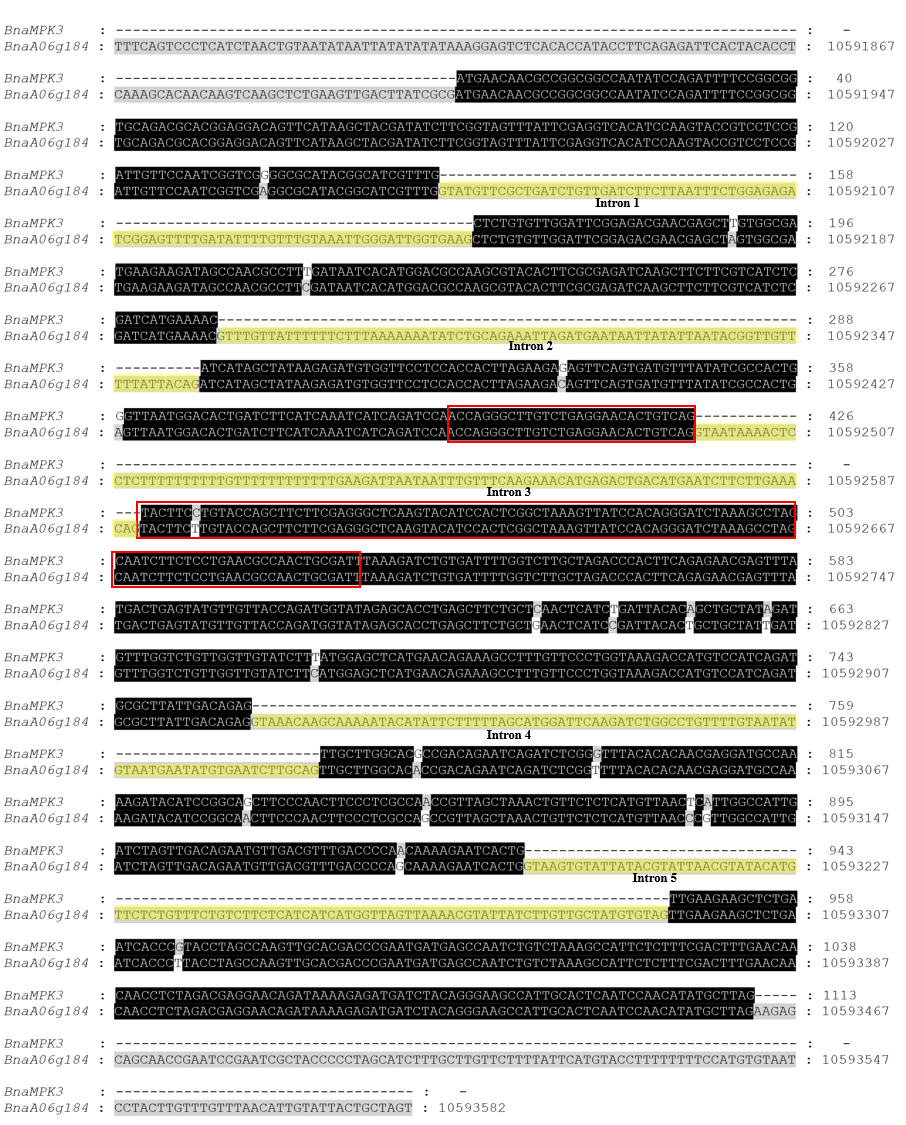

Supplement: FIGURE S4 — Sequence alignment of BnaMPK3 and BnaC03g55440D. The coding sequence of BnaMPK3 was aligned with BnaC03g55440D from a BLAT search. Identical nucleotide bases are shown in black boxes. The intron sequences are shown in the yellow boxes. The RNAi sequences are showed within the red frame. [file Image_4.TIF]

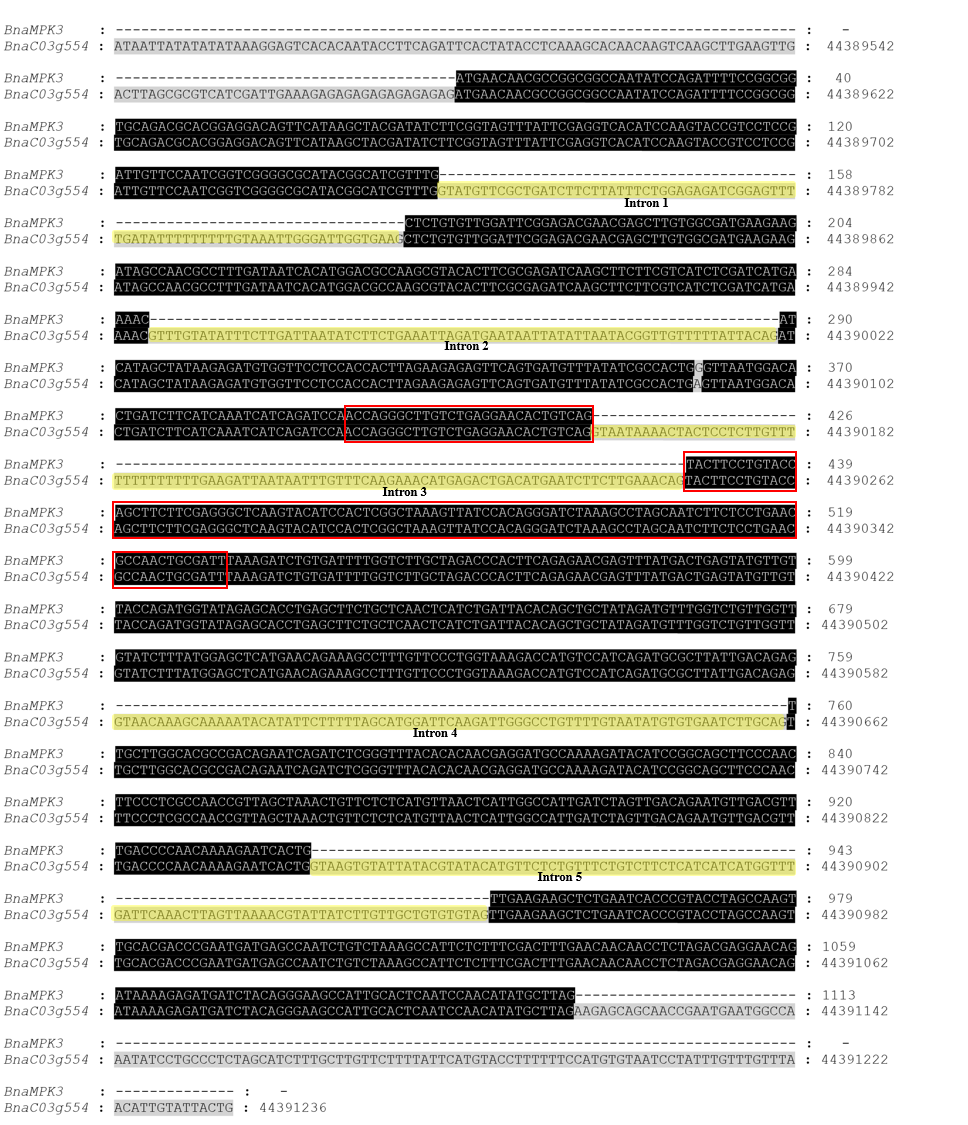

Supplement: FIGURE S5 — Sequence alignment of BnaMPK3 and BnaA06g18440D. The coding sequence of BnaMPK3 was aligned with BnaA06g18440D from a BLAT search. Identical nucleotide bases are shown in black boxes. The intron sequences are shown in the yellow boxes. The RNAi sequences are showed within the red frame. [file Image_5.TIF]

## Supplementary File 2

### Dissociation curves for all amplicons generated by all primer sets

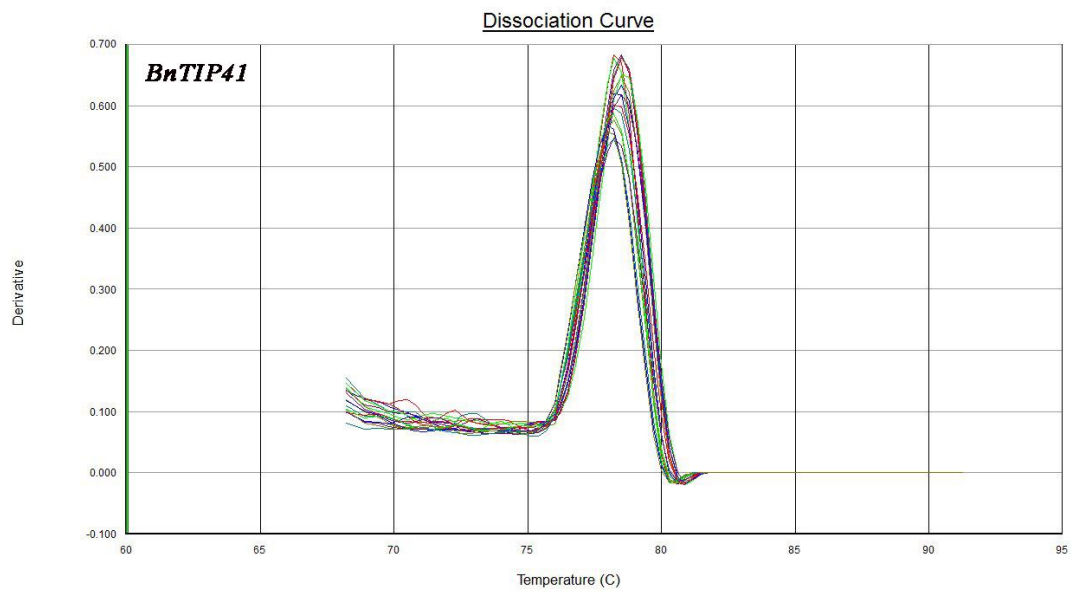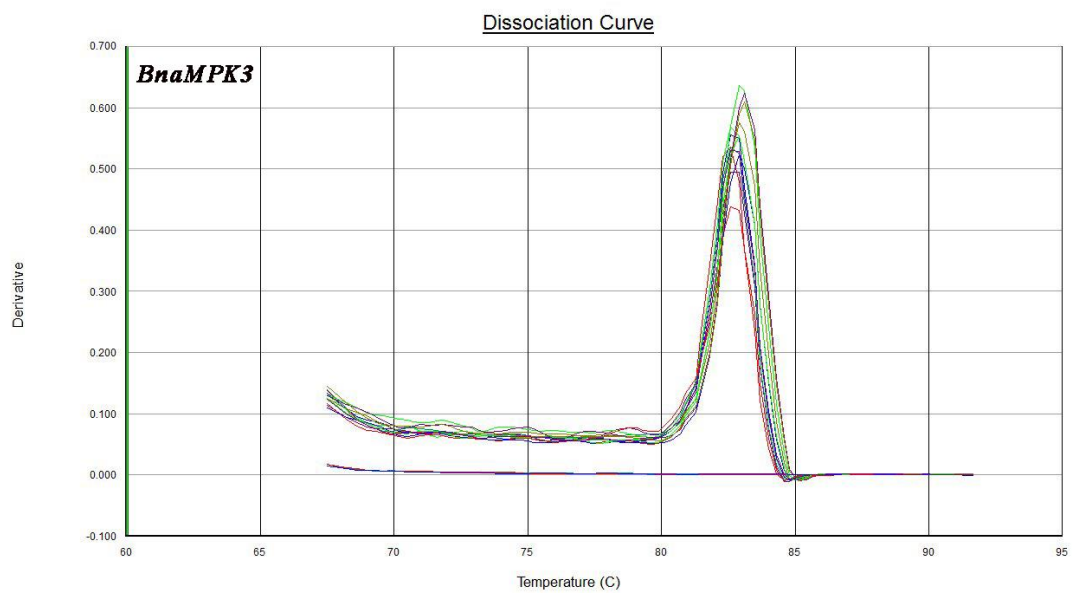

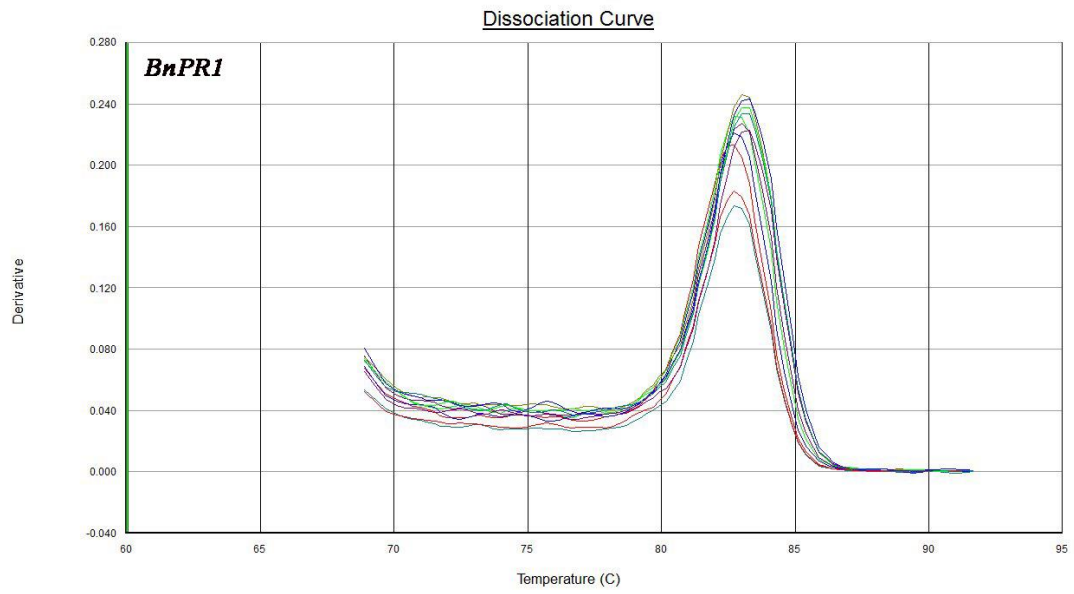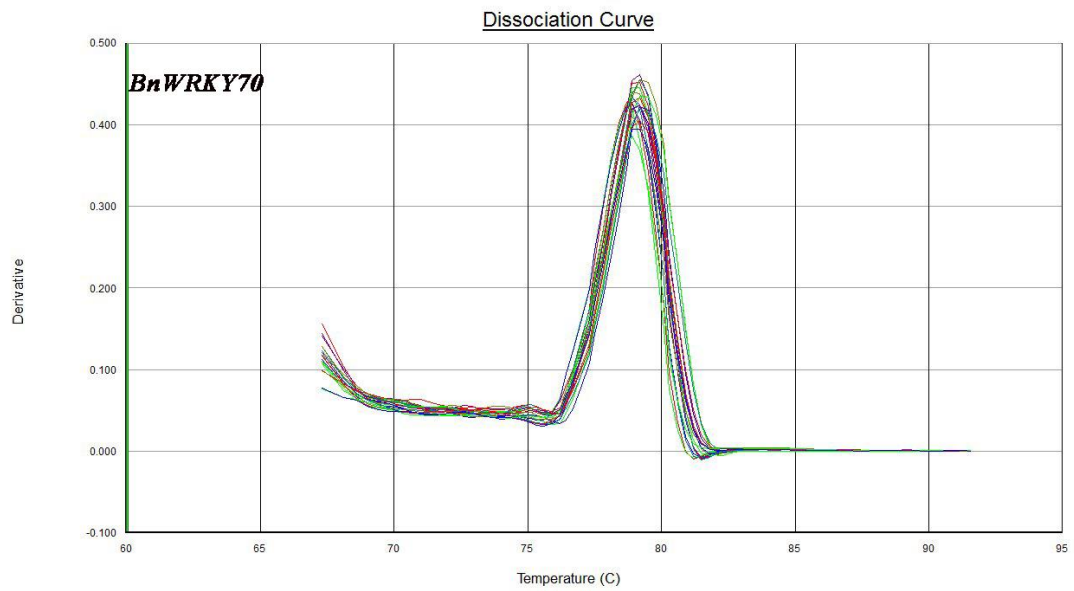

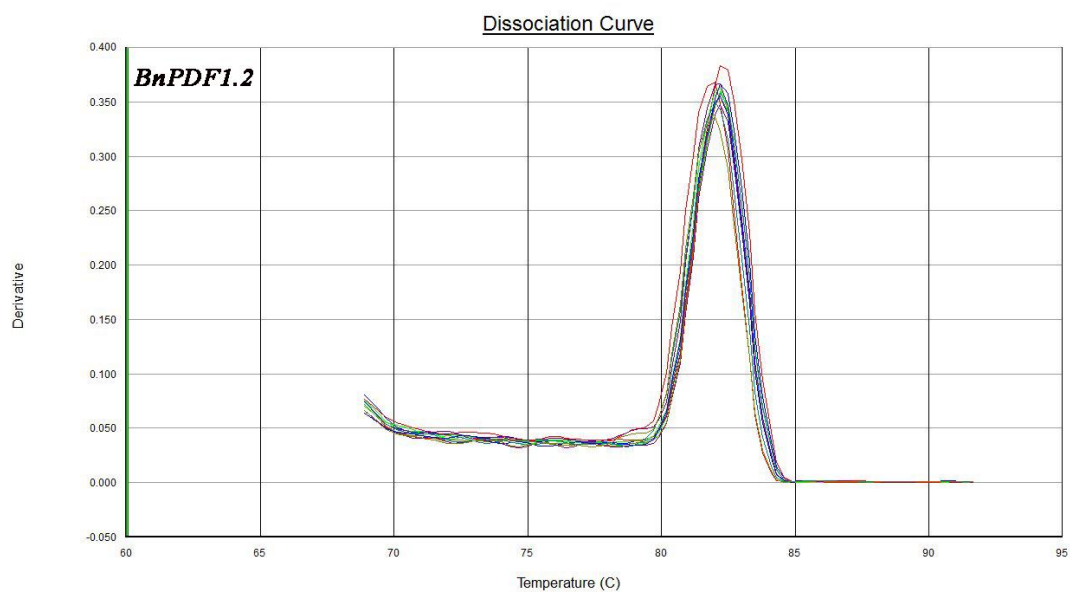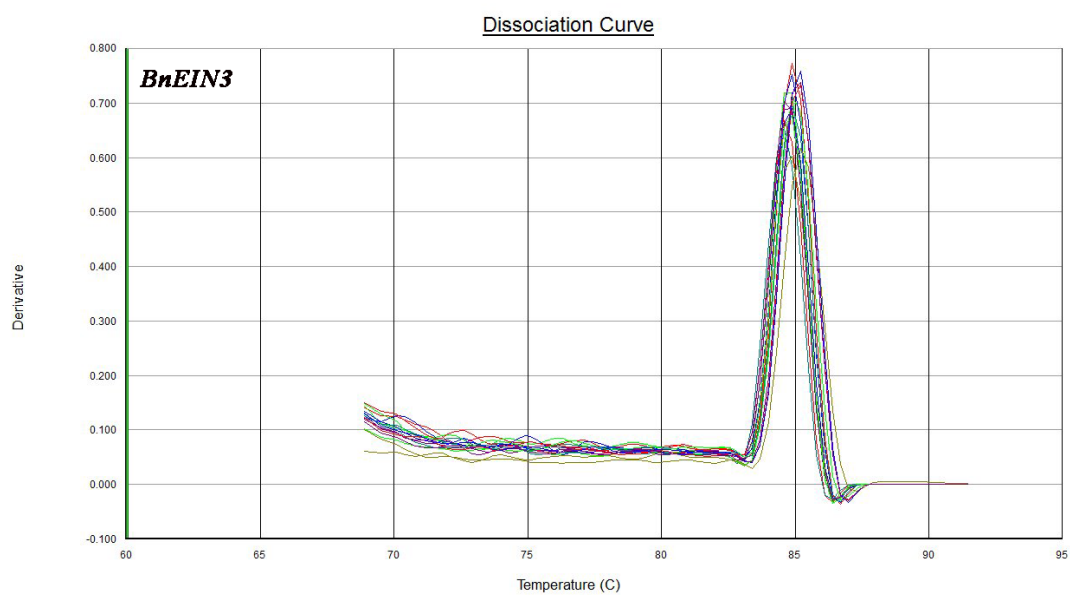

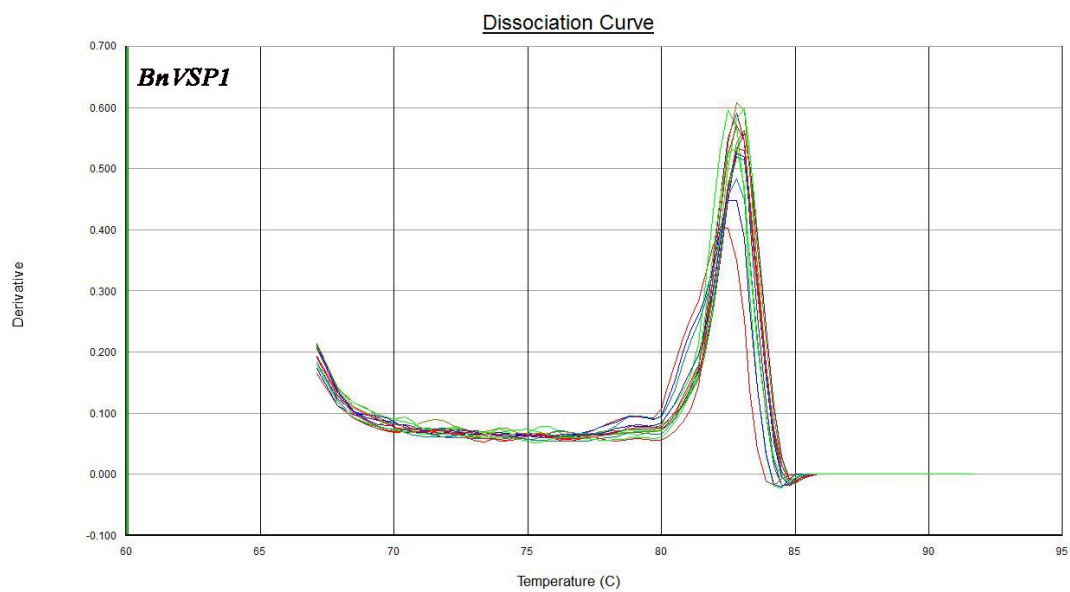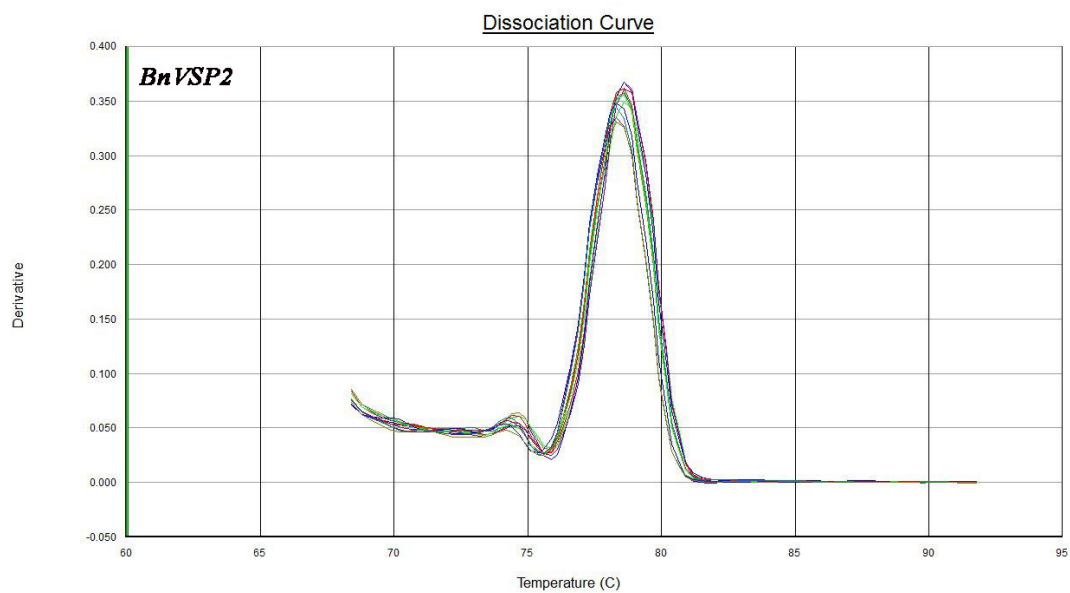

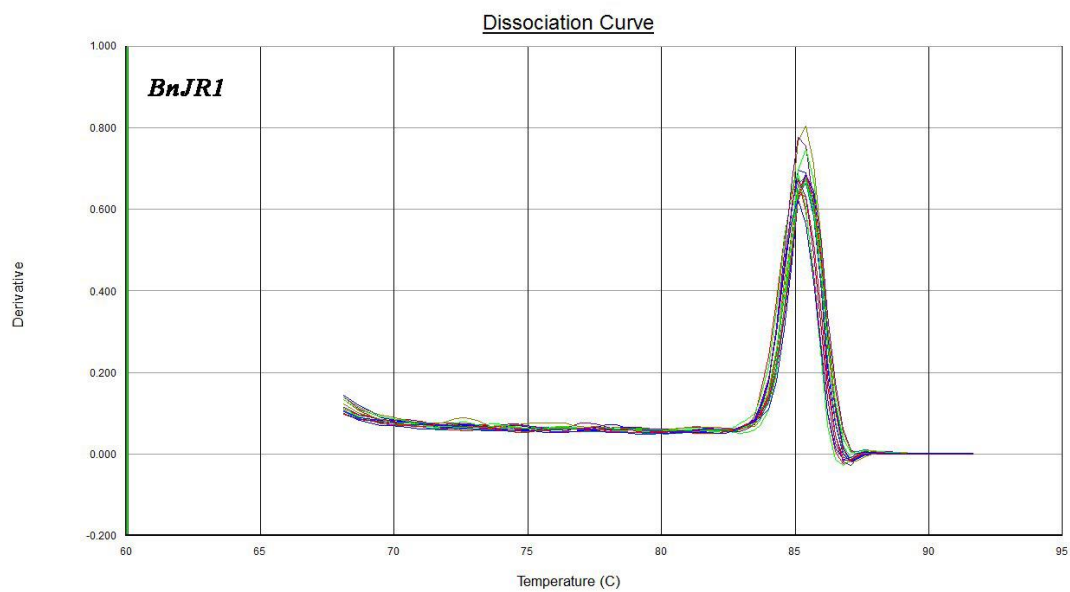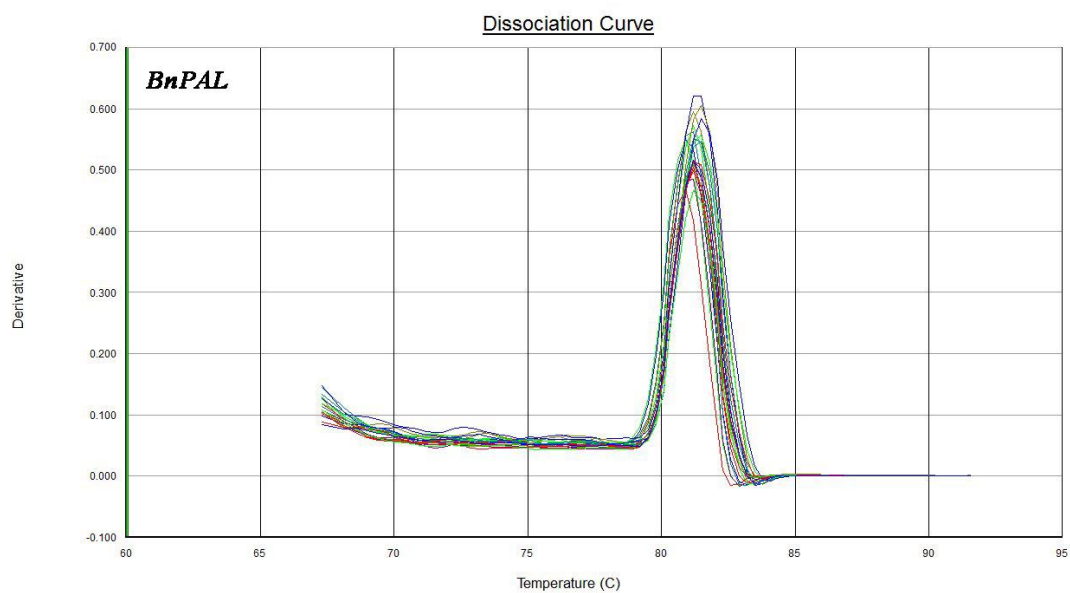

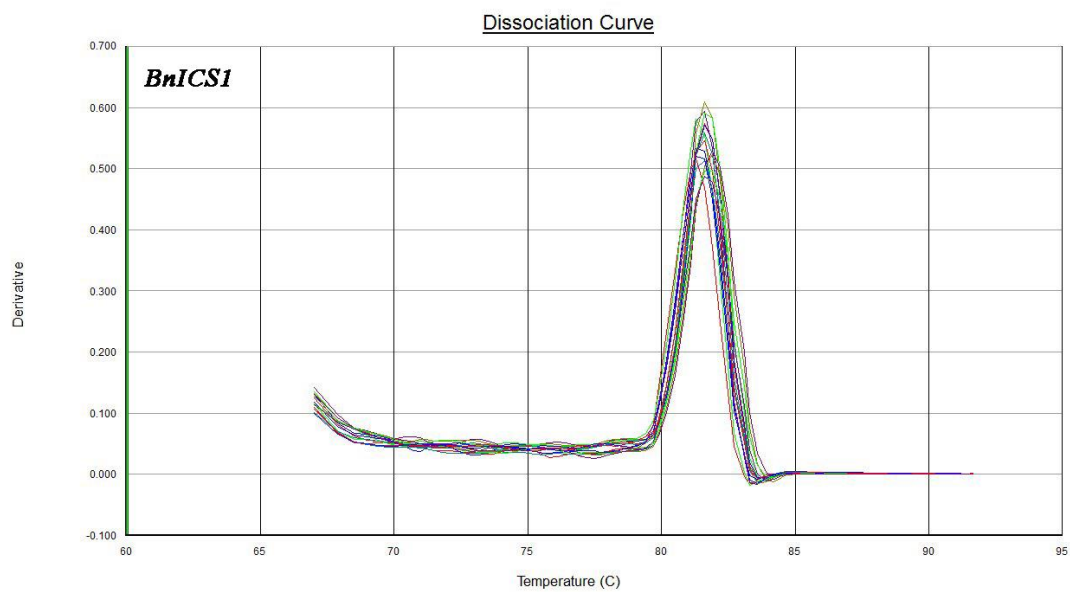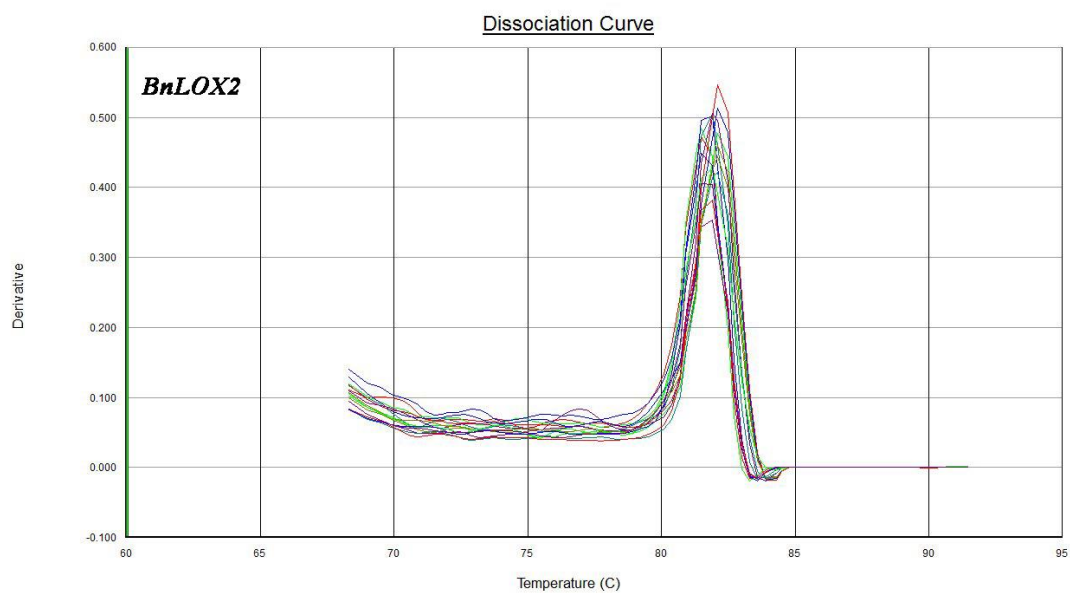

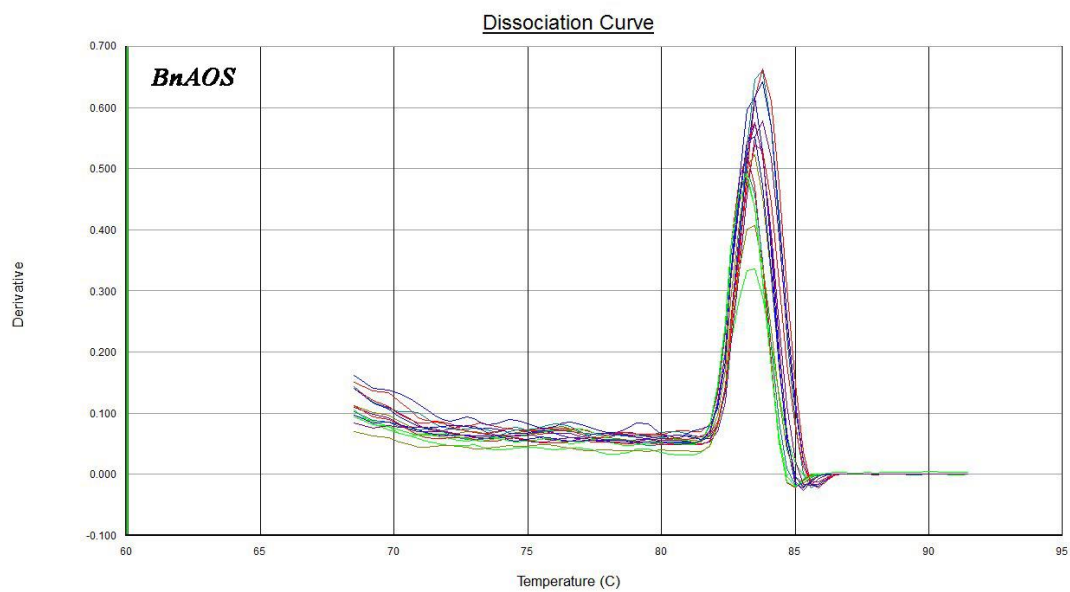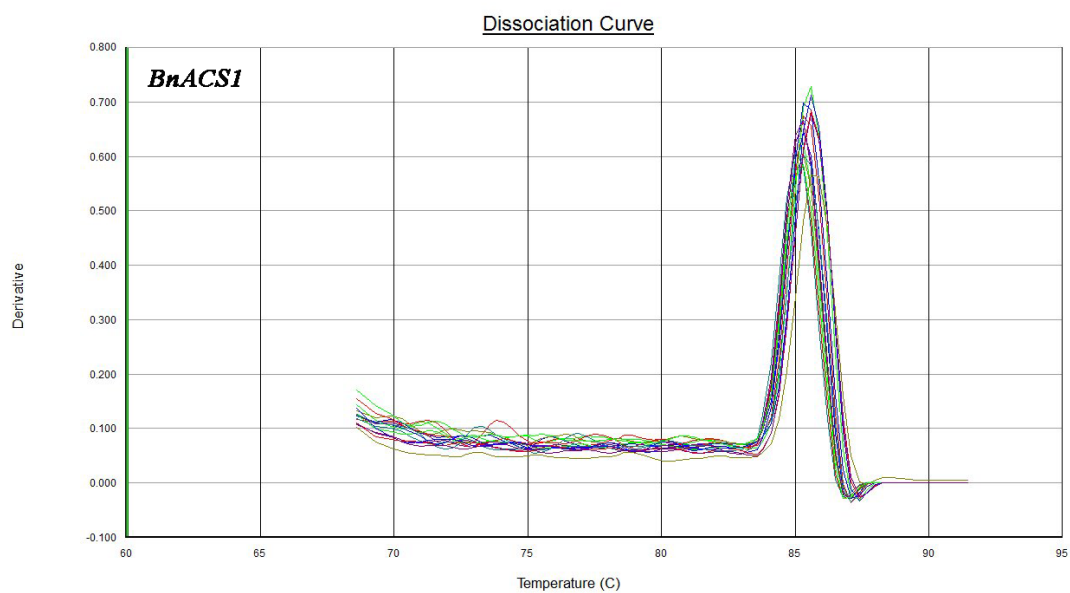

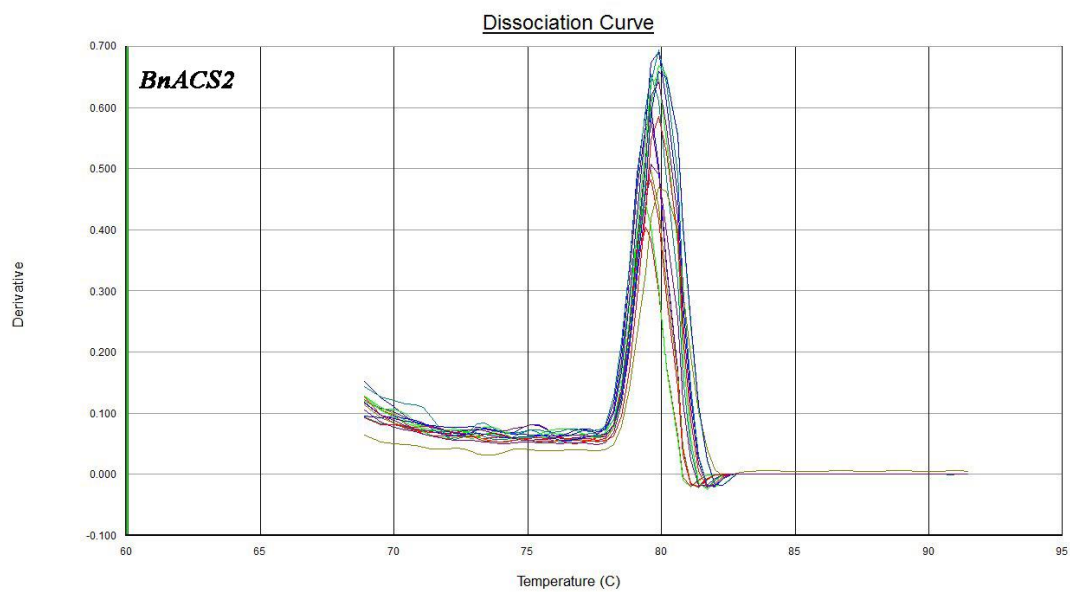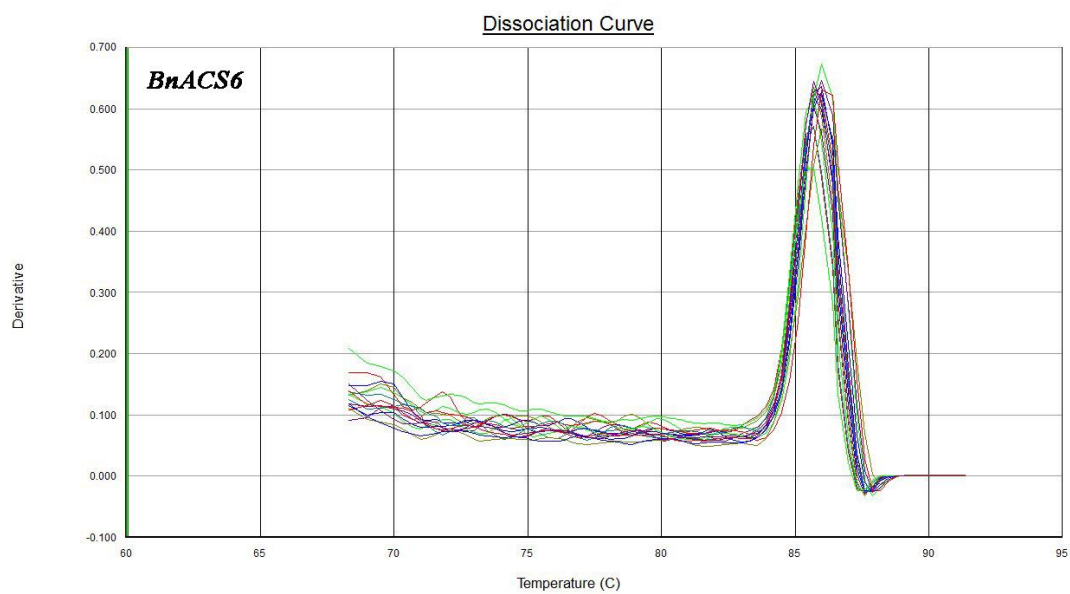

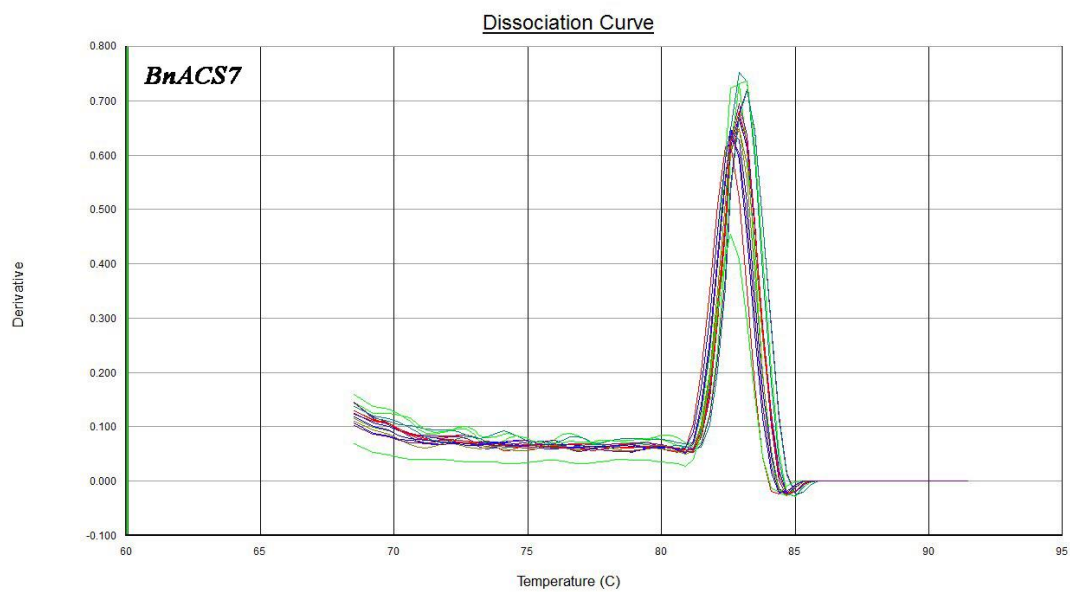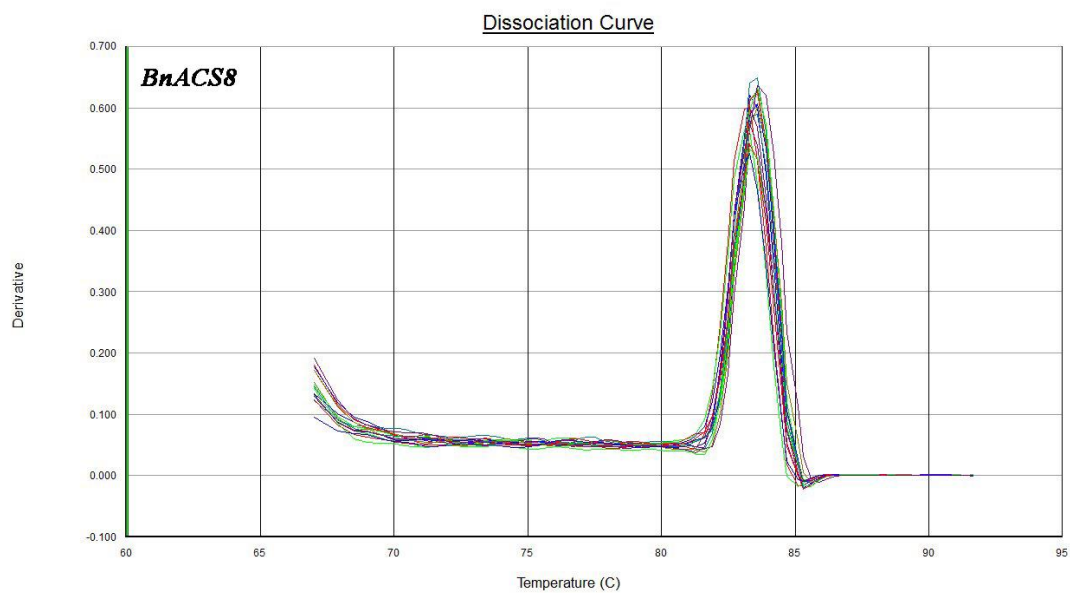

Supplement: FILE S2 — Dissociation curves for all amplicons generated by all primer sets. [file Data_Sheet_2.PDF]
