# Supplementary material for: BnaMPK3 Is a Key Regulator of Defense Responses to the Devastating Plant Pathogen Sclerotinia sclerotiorum in Oilseed Rape
Source: Front Plant Sci. 2019 Feb 8;10:91. doi: 10.3389/fpls.2019.00091 (PMC6376111; doi:10.3389/fpls.2019.00091)
Supplement: TABLE S1 — Primers used for qRT-PCR. [file Table_1.DOC]

Table S1. Primers used for qRT-PCR

| Gene | GenBank Accession | Characteristic | Primer name | Primer sequence |
| --- | --- | --- | --- | --- |
| *BnTIP41* | XM_013842392.2 | TIP41-like Protein  Housekeeping gene | TIP41-F | TGAAGAGCAGATTGATTTGGCT |
|  |  |  | TIP41-R | ACACTCCATTGTCAGCCAGTT |
| *BnaMPK3* | KU363194 |  | MPK3-F | ACCAGGGCTTGTCTGAGGA |
|  |  |  | MPK3-R | AATCGCAGTTGGCGTTCA |
| *BnPR1* | AY623008 | SA-responsive gene | PR1-F | ATGCCAACGCTCACAACCA |
|  |  |  | PR1-R | CACGGGACCTACGCCTACT |
| *BnWRKY70* | EV113862 | SA-responsive gene | WRKY70-F | ACATACATAGGAAACCACACG |
|  |  |  | WRKY70-R | ACTTGGACTATCTTCAGAATGC |
| *BnVSP1* | NM_122387 | JA-responsive gene | VSP1-F | ACCTCTTGGAACTCGGGATTG |
|  |  |  | VSP1-R | GGCTATTCCTAACCTTTGACTTGTA |
| *BnVSP2* | XM_013866303.2 | JA-responsive gene | VSP2-F | CCAACGGGTTCTAACACAAAG |
|  |  |  | VSP2-R | TTCTTGTAGTAGAGTGGATTAGGGA |
| *BnJR1* | NM_001338234.1 | JA-responsive gene | JR1-F | AGGTGATTCTGGTGTTGTTTACG |
|  |  |  | JR1-R | CATAGGTGATGTAGTCTTCAGGAT |
| *BnICS1* | EV225528 | SA biosynthesis gene | ICS1-F | CAAACTCATCATCTTCCCTC |
|  |  |  | ICS1-R | AGCGTGACTTACTAACCAG |
| *BnPAL* | U70666 | SA biosynthesis gene | PAL-F | GCTCACAACCAAGCACGACA |
|  |  |  | PAL-R | TTGCCACATCCAATTCTCACAG |
| *BnLOX2* | AY162143 | JA biosynthesis gene | LOX2-F | TAAAGAGGCGTCGTCCAGTC |
|  |  |  | LOX2-R | ACCAACGGAAGGTAACAAGC |
| *BnAOS* | EV124323 | JA biosynthesis gene | AOS-F | CGCCACCAAAACAACAAAG |
|  |  |  | AOS-R | GGGAGGAAGGAGAGAGGTTG |
| *BnPDF1.2* | AY884023 | SA/JA-responsive gene | PDF1.2-F | CATCACCCTTCTCTTCGCTGC |
|  |  |  | PDF1.2-R | ATGTCCCACTTGACCTCTCGC |
| *BnEIN3* | XM_013893836.2 | ET-responsive gene | EDS1-F | CGAGACAGCGGCTTACCCTA |
|  |  |  | EDS1-R | GCAGTTGACTTTCCGTGACCAT |
| *BnACS1* | XM_013863169.2 | ET biosynthesis gene | ACS1-F | GCTGTGGCGTGTGATCGTCC |
|  |  |  | ACS1-R | TTATATCTGTGCACGAACAAGCGGAG |
| *BnACS2* | XM_013810867.2 | ET biosynthesis gene | ACS2-F | GGTGGTCAAAGACTTAGATAG |
|  |  |  | ACS2-R | ACCGAGTCGTTGTAAGAATA |
| *BnACS6* | XM_013886694.2 | ET biosynthesis gene | ACS6-F | AGCAAACTACGGTTGGCTGAAAG |
|  |  |  | ACS6-R | TCCTCTCTAGGGCGGTCTCC |
| *BnACS7* | XM_022692758.1 | ET biosynthesis gene | ACS7-F | CGAGCAGGCTTACCAAAC |
|  |  |  | ACS7-R | AAACTCTTCATCCGACAAC |
| *BnACS8* | XM_013799555.2 | ET biosynthesis gene | ACS8-F | GTGCTACTTCGGCTAACG |
|  |  |  | ACS8-R | GCTTGCTTGTAGGCTTCT |
