# Supplementary material for: BnaMPK3 Is a Key Regulator of Defense Responses to the Devastating Plant Pathogen Sclerotinia sclerotiorum in Oilseed Rape
Source: Front Plant Sci. 2019 Feb 8;10:91. doi: 10.3389/fpls.2019.00091 (PMC6376111; doi:10.3389/fpls.2019.00091)
Supplement: FILE S1 — The efficiencies (E) of the primer-specific polymerase chain reaction (PCR) amplifications and variation of ΔCT (CtTarget - CtTIP41) with template dilution. [file Data_Sheet_1.PDF]

Supplementary File 1

The efficiencies (E) of the primer-specific polymerase chain reaction (PCR) amplifications and variation of Δ*C*T（Ct*Target*-Ct*TIP41*）with template dilution

Gene	R^2	Slope	E
BnTIP41	0.9998	-3.3915	97.18%
BnMPK3	0.9998	-3.3956	97.01%
BnPR1	0.9995	-3.3385	99.31%
BnWRKY70	0.9996	-3.3506	98.82%
BnRDF1.2	0.9983	-3.3291	99.70%
BnEIN3	0.9997	-3.3276	99.76%
BnVSP1	0.9997	-3.3514	98.78%
BnVSP2	0.9999	-3.3414	99.19%
BnJR1	1	-3.4384	95.36%
BnPAL	0.9997	-3.3658	98.20%
BnICS1	0.9998	-3.3368	99.38%
BnLOX2	0.9999	-3.3608	98.40%
BnAOS	0.9991	-3.4404	95.28%
BnACS1	0.9997	-3.3708	98.00%
BnACS2	0.9996	-3.3688	98.08%
BnACS6	0.9999	-3.4091	96.49%
BnACS7	0.9995	-3.3591	98.47%
BnACS8	0.9995	-3.3644	98.26%

$E=10^{(1/-slope)}-1$

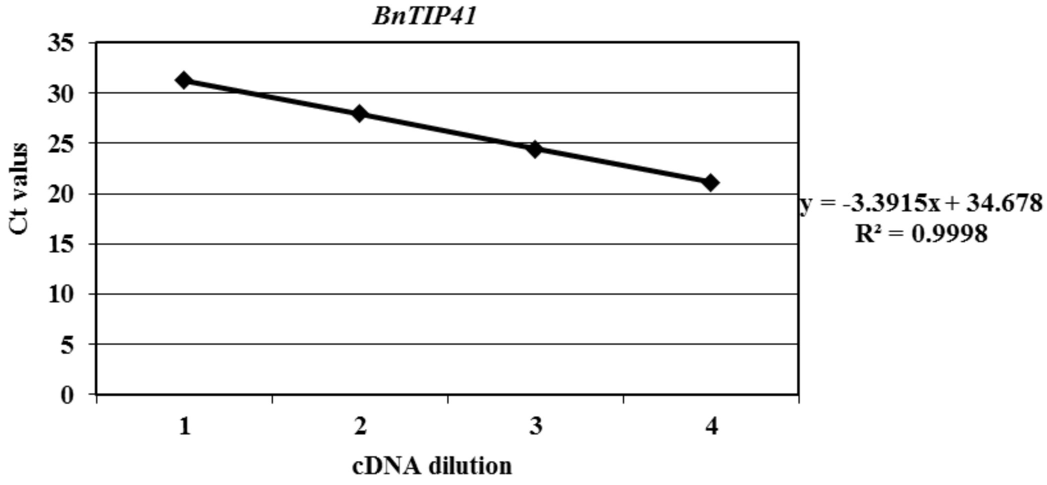

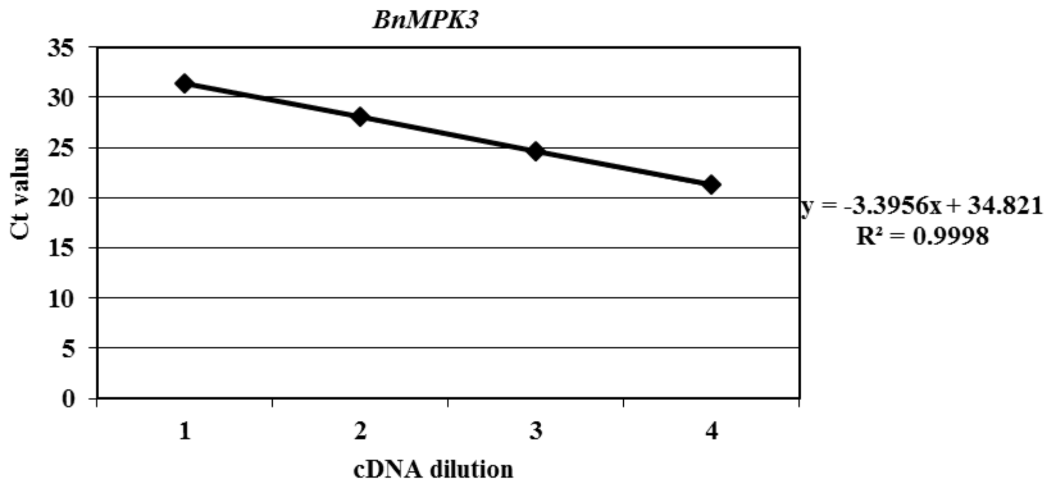
BnMPK3
Ct valus
35
30
25
20
15
10
5
0
1
2
3
4
cDNA dilution
y = -3.3956x + 34.821
R² = 0.9998

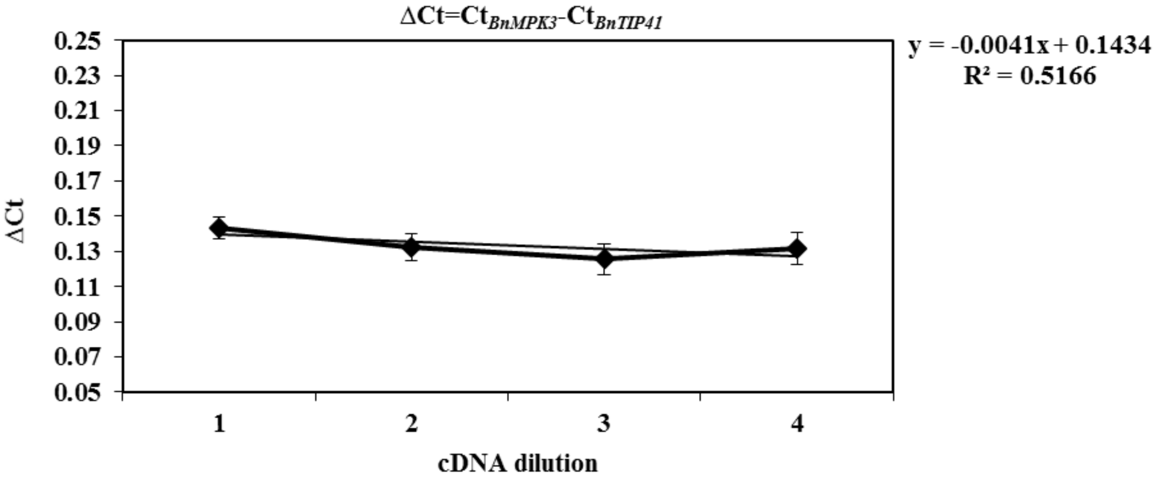
ΔCt=Ct BnMPK3-Ct BnTIP41
ΔCt
0.25
0.23
0.21
0.19
0.17
0.15
0.13
0.11
0.09
0.07
0.05
1
2
3
4
cDNA dilution
y = -0.0041x + 0.1434
R² = 0.5166

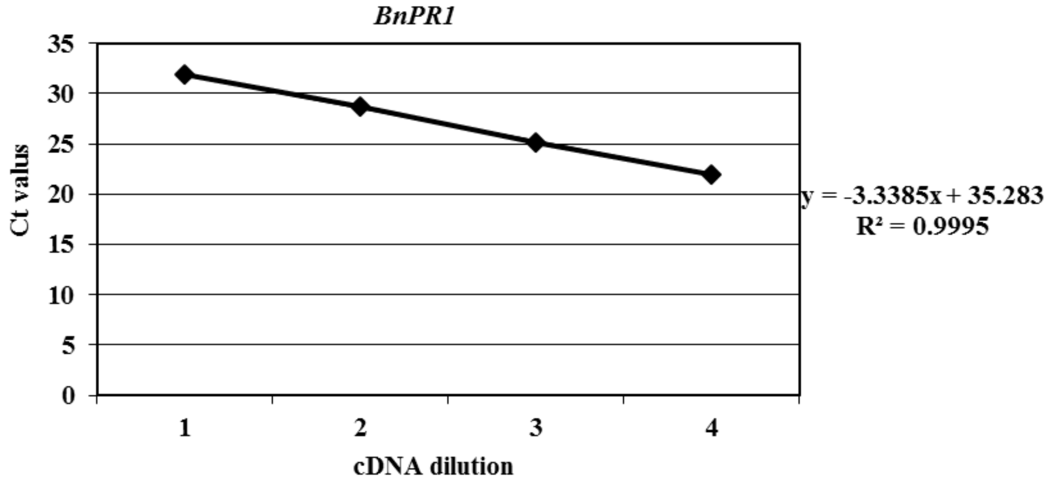
BnPR1
Ct valus
35
30
25
20
15
10
5
0
1
2
3
4
cDNA dilution
y = -3.3385x + 35.283
R² = 0.9995

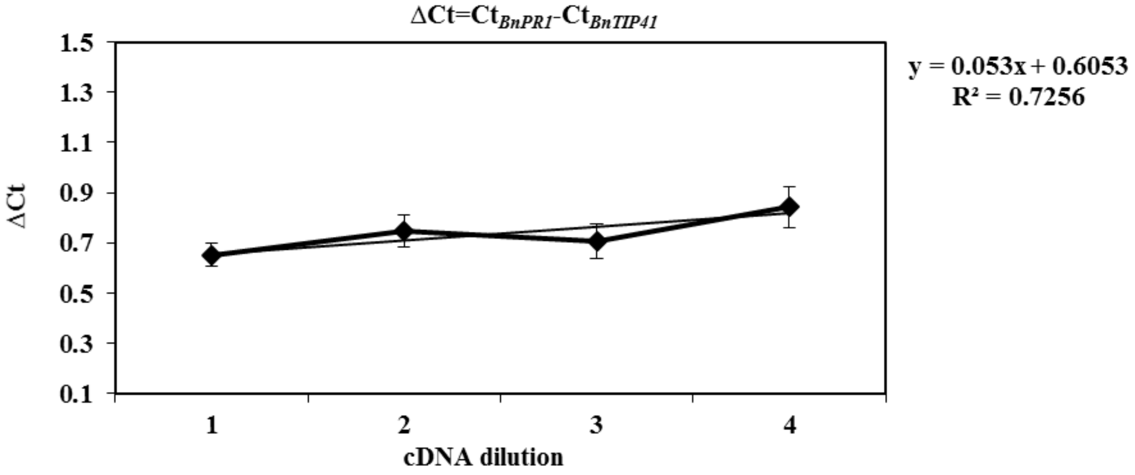
ΔCt=Ct BnPR1-Ct BnTIP41
y = 0.053x + 0.6053
R² = 0.7256
ΔCt
1.5
1.3
1.1
0.9
0.7
0.5
0.3
0.1
1
2
3
4
cDNA dilution

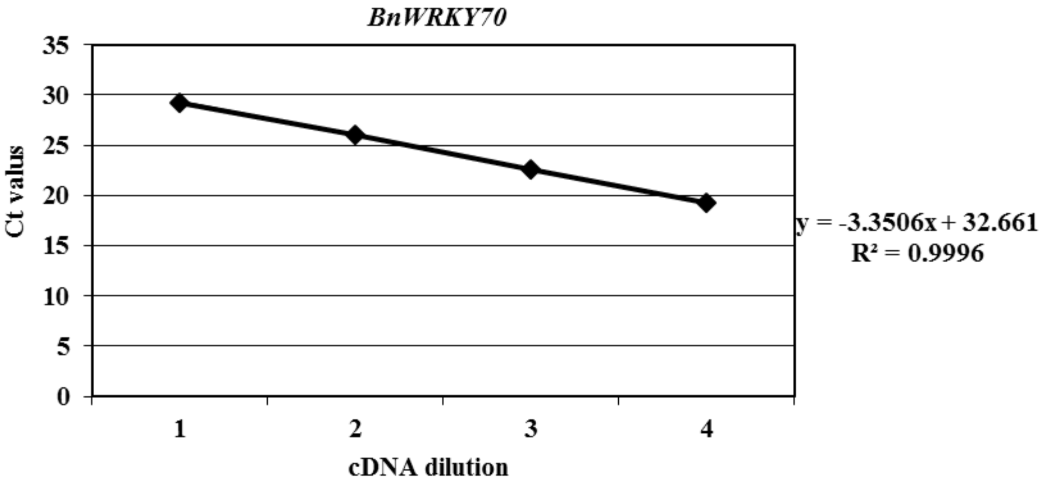
BnWRKY70
Ct valus
35
30
25
20
15
10
5
0
y = -3.3506x + 32.661
R² = 0.9996
1
2
3
4
cDNA dilution

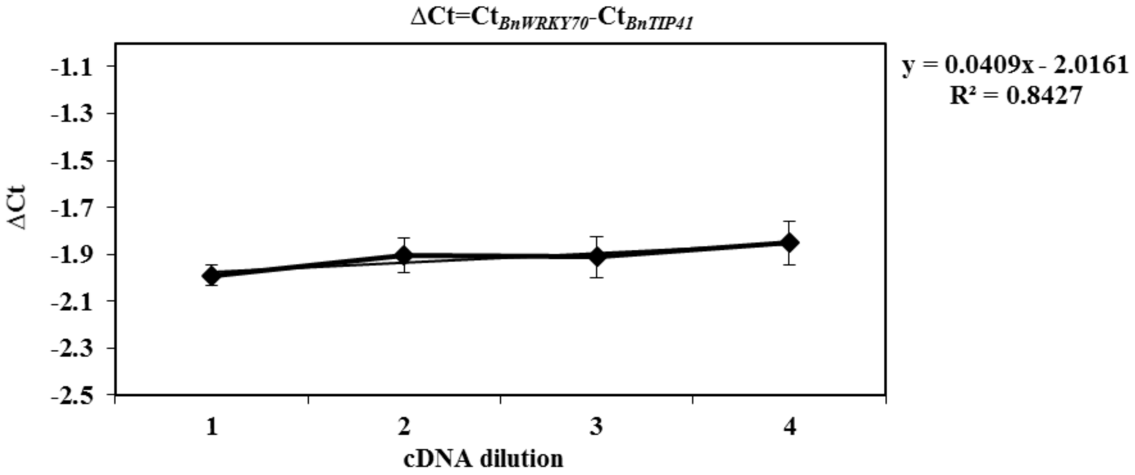
ΔCt=Ct BnWRKY70-Ct BnTIP41
y = 0.0409x - 2.0161
R² = 0.8427
ΔCt
-1.1
-1.3
-1.5
-1.7
-1.9
-2.1
-2.3
-2.5
1
2
3
4
cDNA dilution

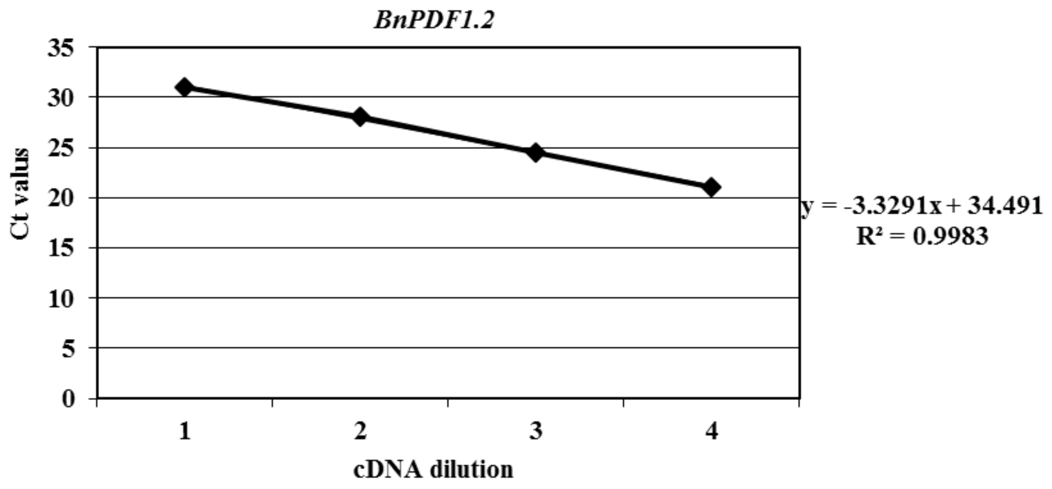

BnPDF1.2
Ct valus
35
30
25
20
15
10
5
0
1
2
3
4
cDNA dilution
y = -3.3291x + 34.491
R² = 0.9983

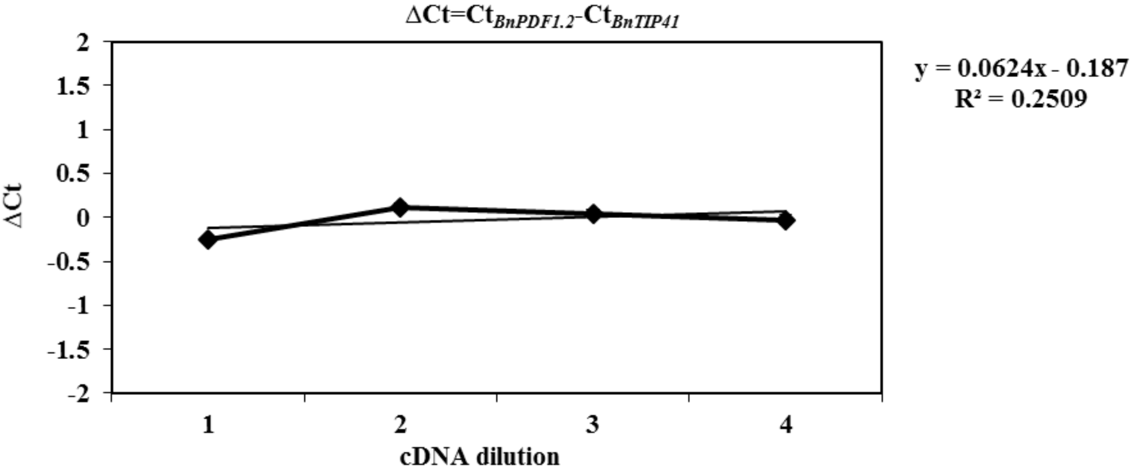

ΔCt=Ct BnPDF1.2-Ct BnTIP41
ΔCt
2
1.5
1
0.5
0
-0.5
-1
-1.5
-2
1
2
3
4
cDNA dilution
y = 0.0624x - 0.187
R² = 0.2509

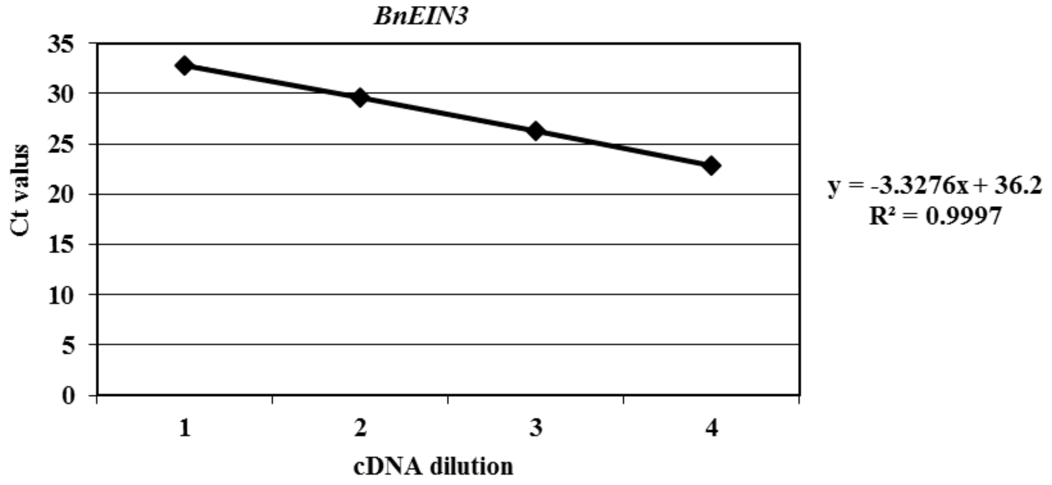

BnEIN3
Ct valus
35
30
25
20
15
10
5
0
1
2
3
4
cDNA dilution
y = -3.3276x + 36.2
R² = 0.9997

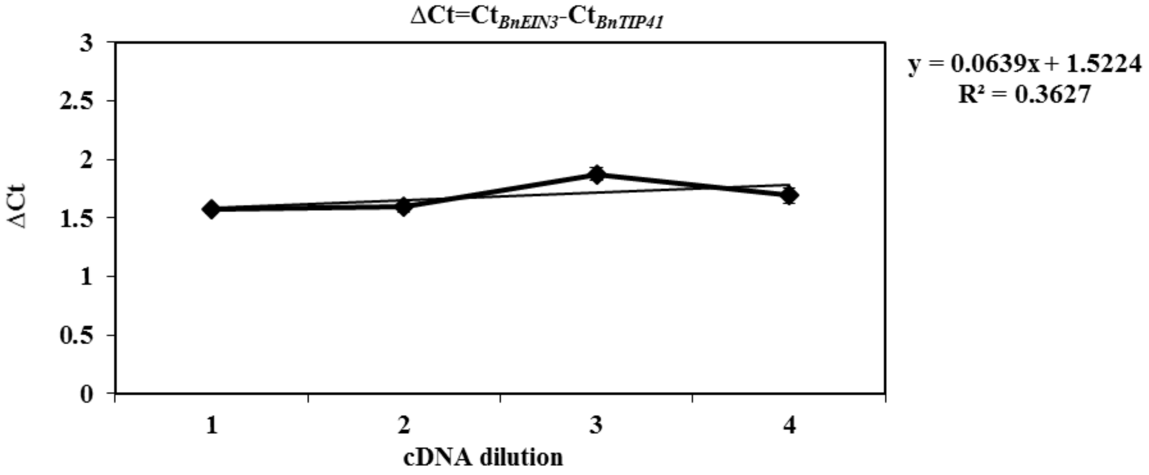

ΔCt=Ct_BnEIN3-Ct_BnTIP41
y = 0.0639x + 1.5224
R² = 0.3627
ΔCt
3
2.5
2
1.5
1
0.5
0
1
2
3
4
cDNA dilution

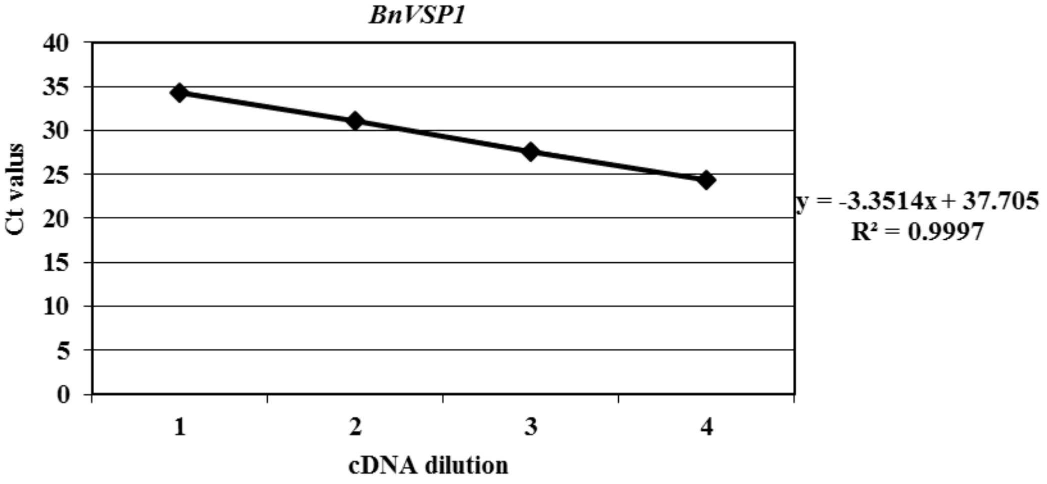

BnVSP1
Ct valus
40
35
30
25
20
15
10
5
0
1
2
3
4
cDNA dilution
y = -3.3514x + 37.705
R² = 0.9997

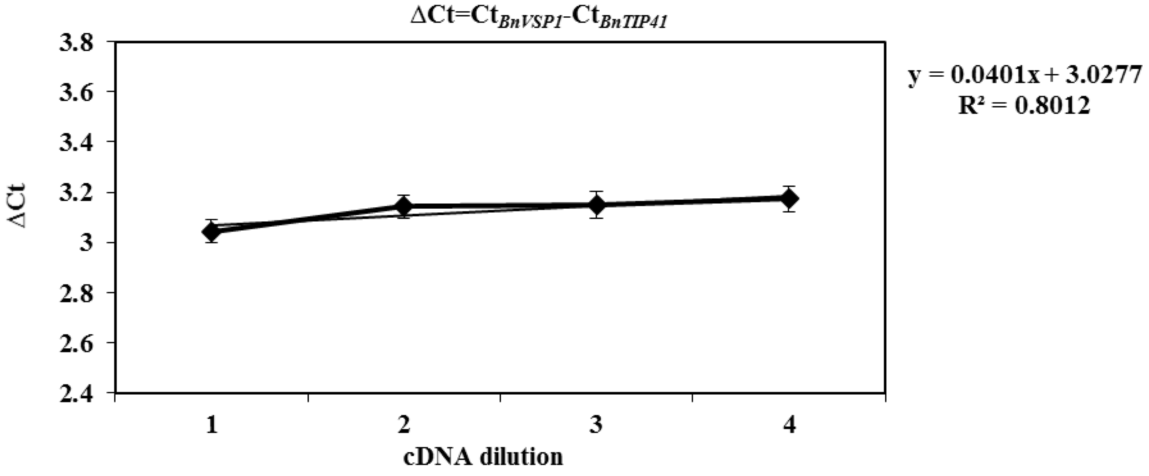

ΔCt=Ct_BnVSP1-Ct_BnTIP41
y = 0.0401x + 3.0277
R² = 0.8012
ΔCt
3.8
3.6
3.4
3.2
3
2.8
2.6
2.4
1
2
3
4
cDNA dilution

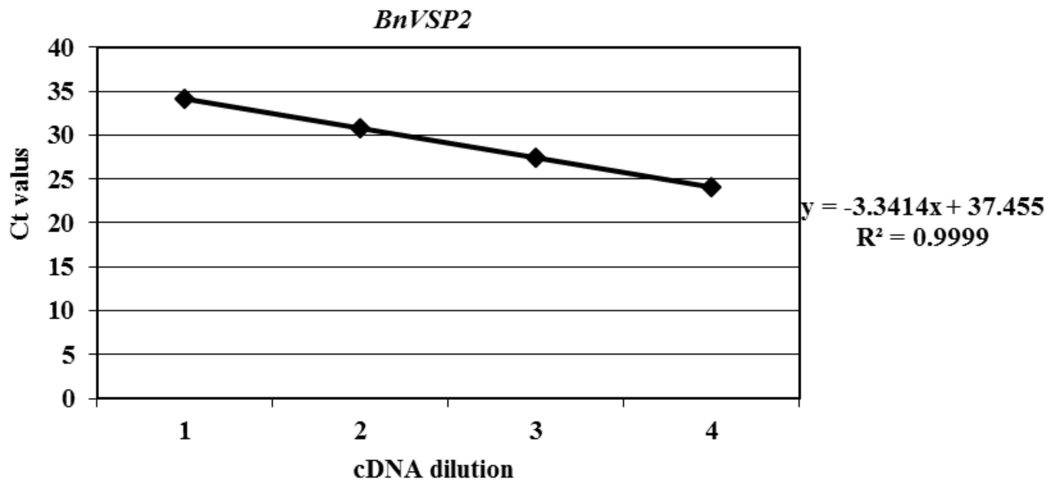
BnVSP2
Ct valus
40
35
30
25
20
15
10
5
0
1
2
3
4
cDNA dilution
y = -3.3414x + 37.455
R² = 0.9999

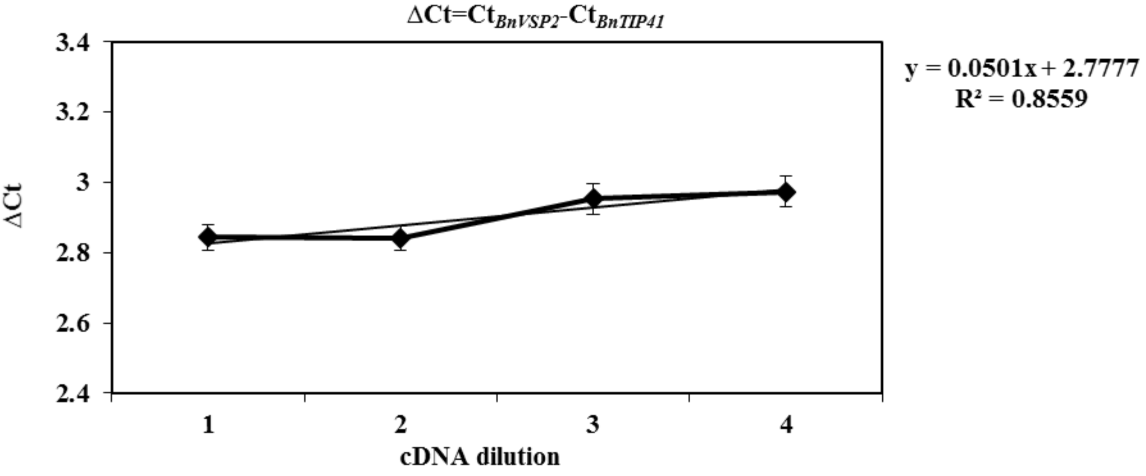
ΔCt=Ct_BnVSP2-Ct_BnTIP41
ΔCt
3.4
3.2
3
2.8
2.6
2.4
1
2
3
4
cDNA dilution
y = 0.0501x + 2.7777
R² = 0.8559

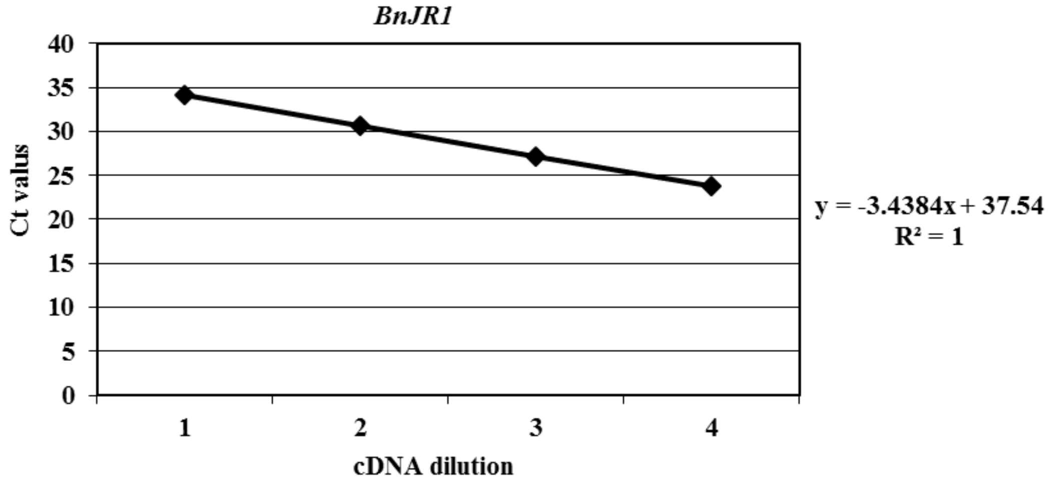
BnJR1
Ct valus
40
35
30
25
20
15
10
5
0
1
2
3
4
cDNA dilution
y = -3.4384x + 37.54
R² = 1

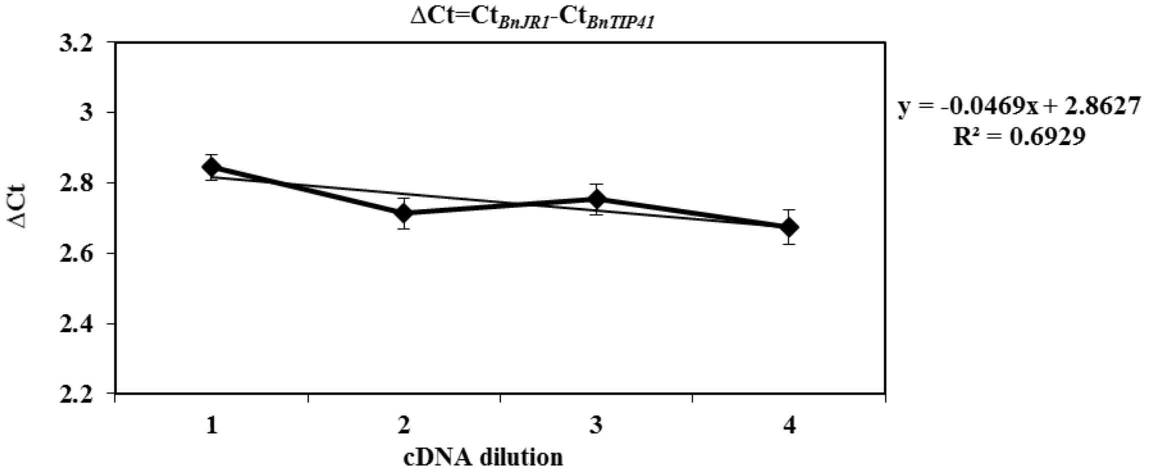
ΔCt=Ct BnJR1-Ct BnTIP41
ΔCt
3.2
3
2.8
2.6
2.4
2.2
1
2
3
4
cDNA dilution
y = -0.0469x + 2.8627
R² = 0.6929

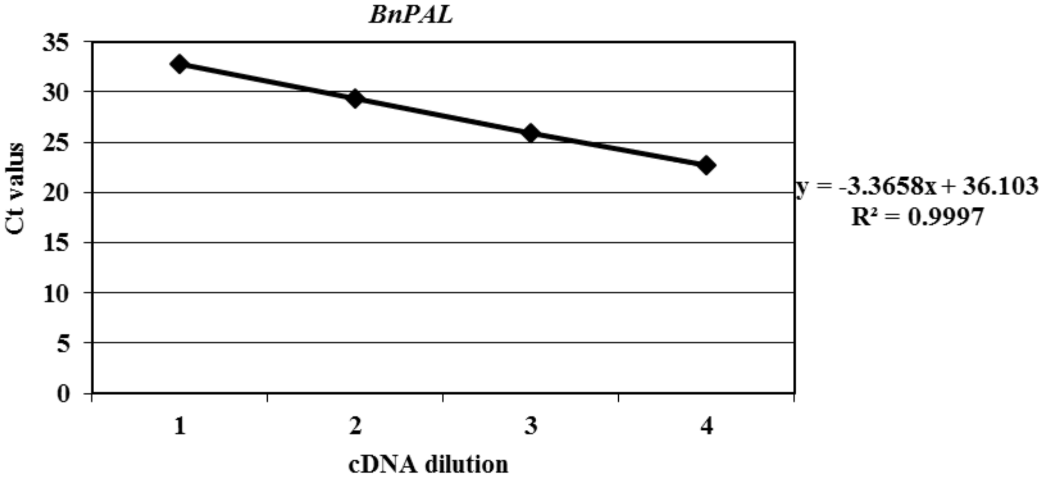
BnPAL
Ct valus
35
30
25
20
15
10
5
0
1
2
3
4
cDNA dilution
y = -3.3658x + 36.103
R² = 0.9997

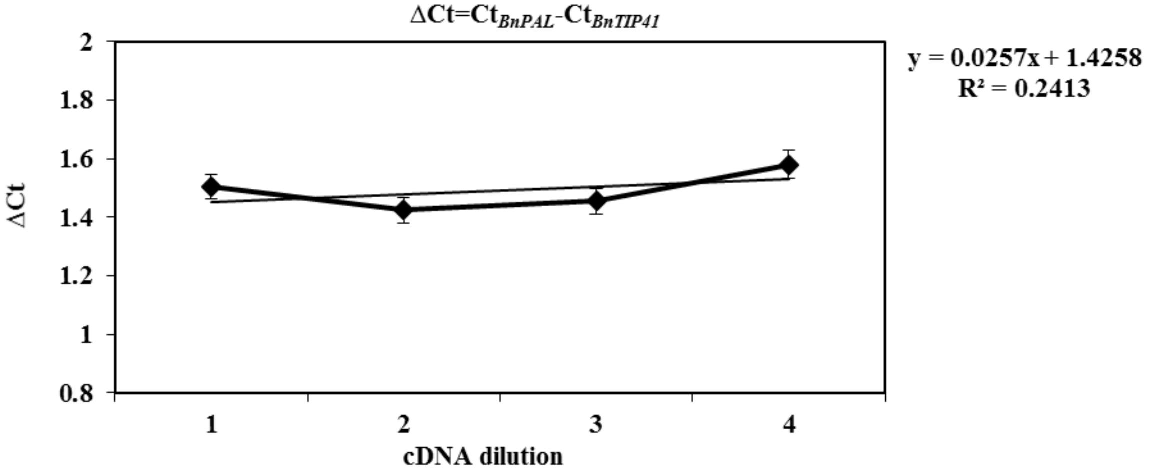
ΔCt=Ct BnPAL-Ct BnTIP41
ΔCt
2
1.8
1.6
1.4
1.2
1
0.8
1
2
3
4
cDNA dilution
y = 0.0257x + 1.4258
R² = 0.2413

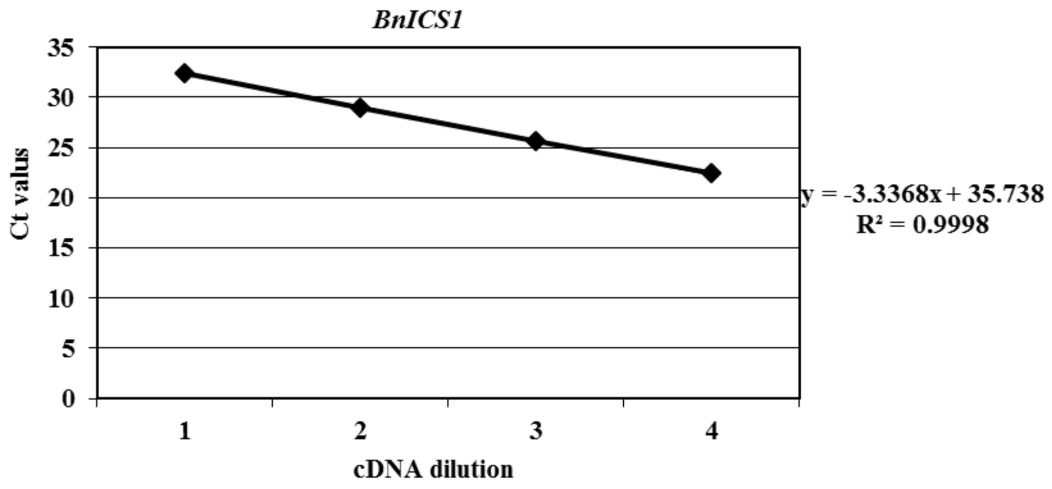
BnICS1
Ct valus
cDNA dilution
y = -3.3368x + 35.738
R² = 0.9998
0
5
10
15
20
25
30
35
1
2
3
4

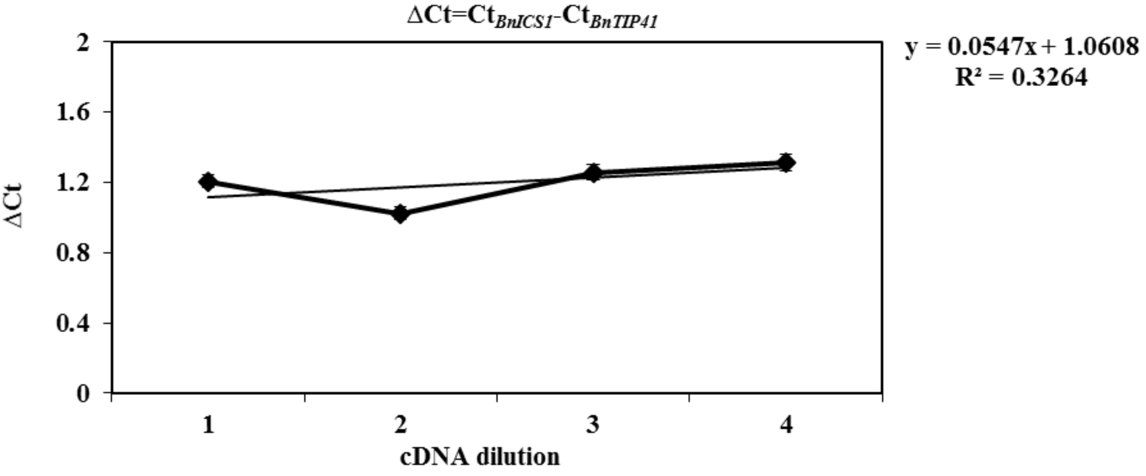
ΔCt=Ct BnICS1 -Ct BnTIP41
ΔCt
cDNA dilution
y = 0.0547x + 1.0608
R² = 0.3264
0
0.4
0.8
1.2
1.6
2
1
2
3
4

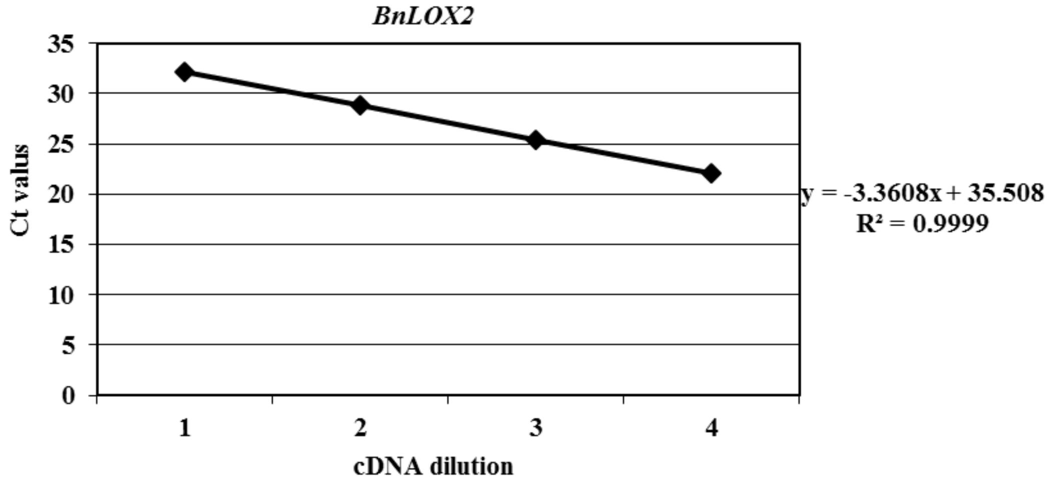
BnLOX2
Ct valus
cDNA dilution
y = -3.3608x + 35.508
R² = 0.9999
0
5
10
15
20
25
30
35
1
2
3
4

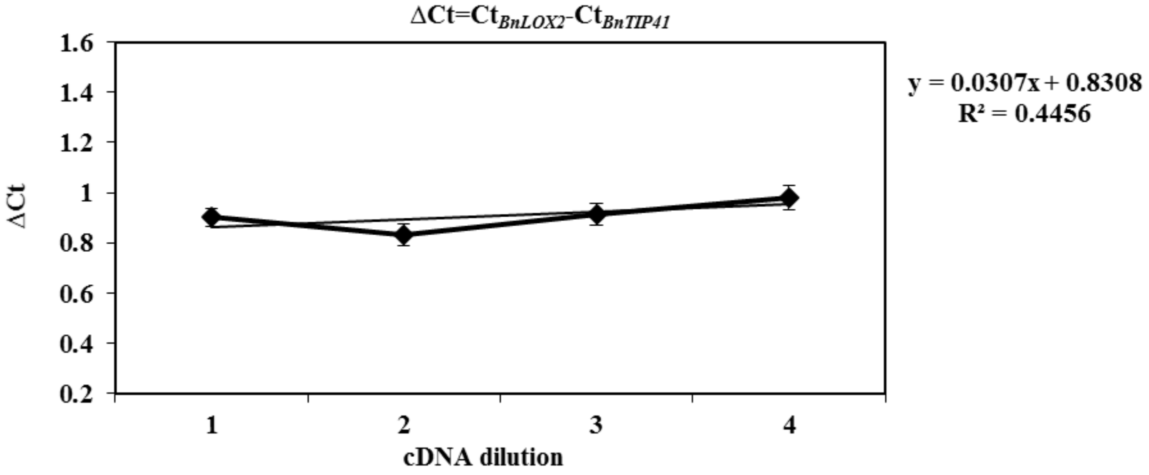
ΔCt=Ct BnLOX2-Ct BnTIP41
1.6
1.4
1.2
1
0.8
0.6
0.4
0.2
ΔCt
1
2
3
4
cDNA dilution
y = 0.0307x + 0.8308
R² = 0.4456

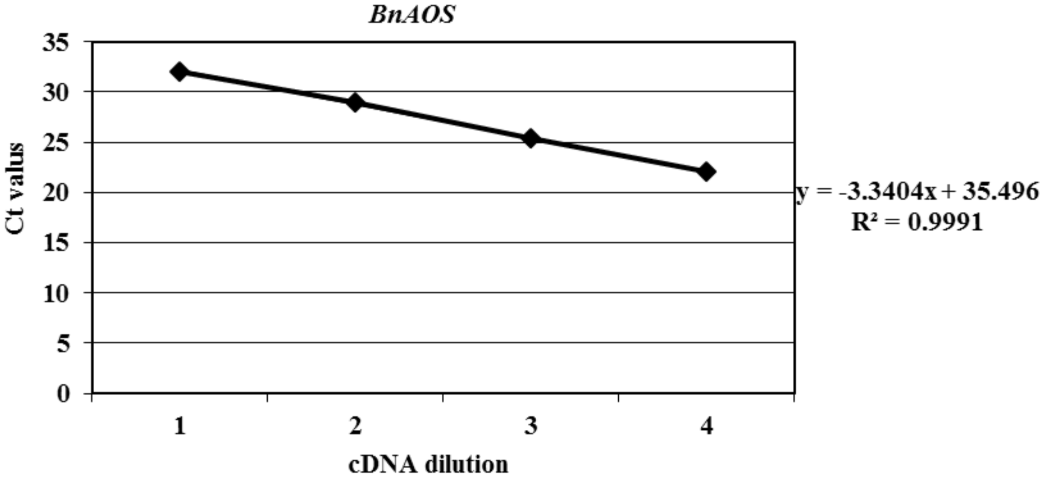
BnAOS
35
30
25
20
15
10
5
0
Ct valus
1
2
3
4
cDNA dilution
y = -3.3404x + 35.496
R² = 0.9991

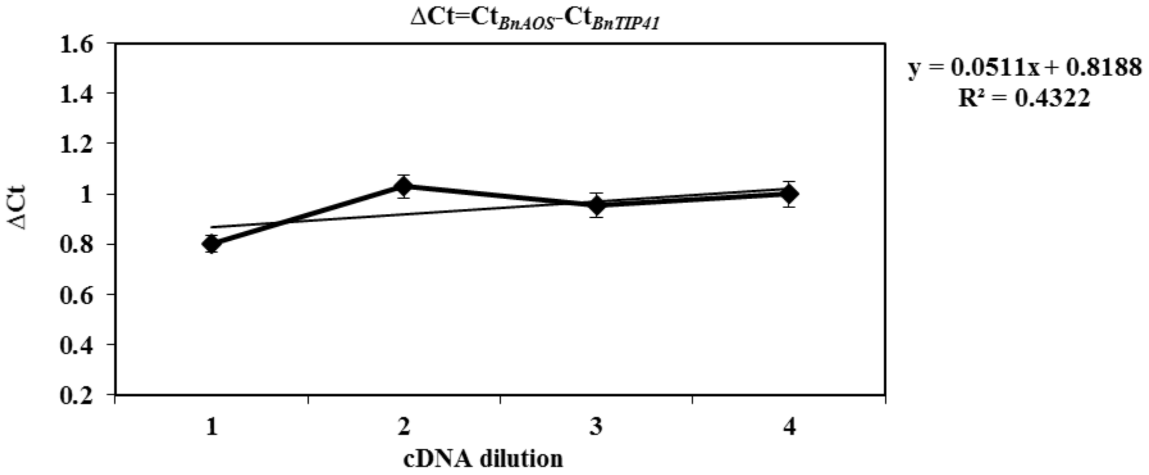
ΔCt=Ct BnAOS-Ct BnTIP41
1.6
1.4
1.2
1
0.8
0.6
0.4
0.2
ΔCt
1
2
3
4
cDNA dilution
y = 0.0511x + 0.8188
R² = 0.4322

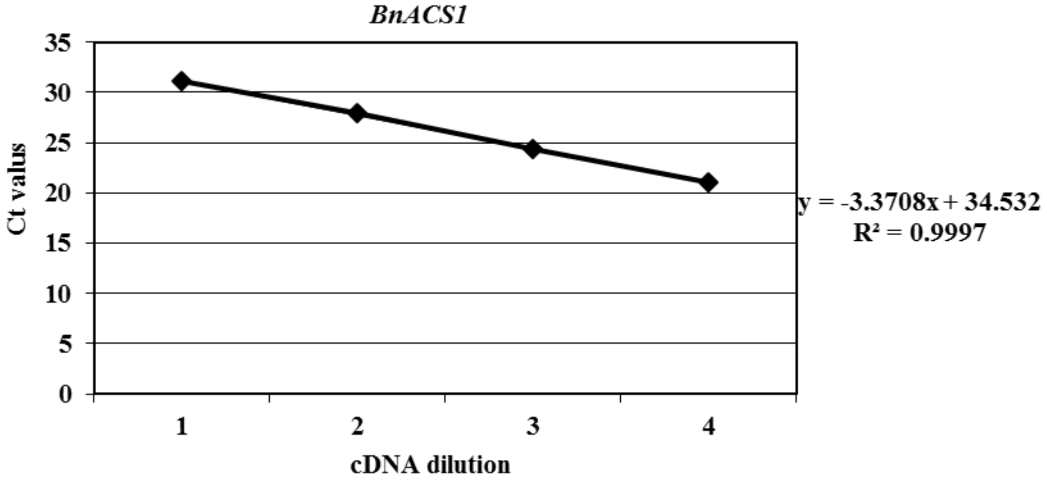
BnACS1
35
30
25
20
15
10
5
0
Ct valus
1
2
3
4
cDNA dilution
y = -3.3708x + 34.532
R² = 0.9997

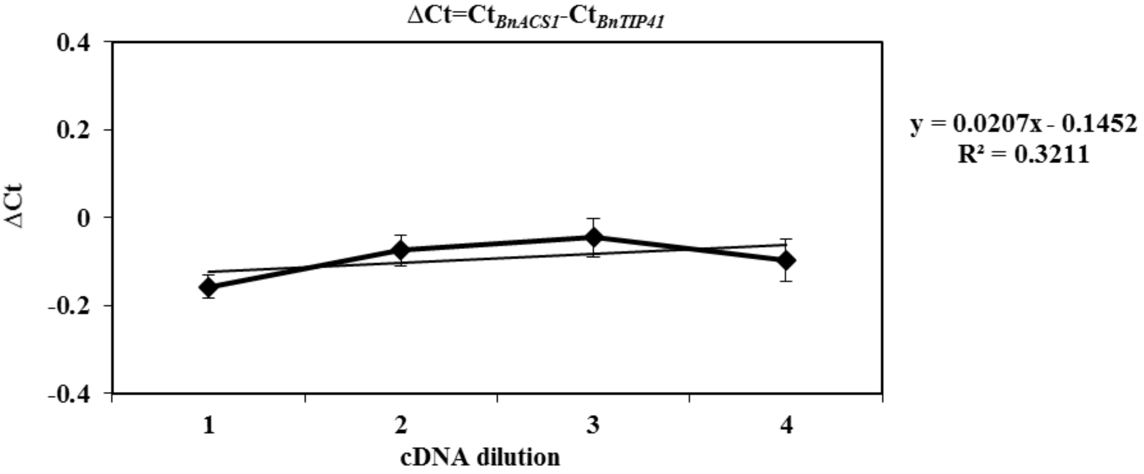
ΔCt=Ct$_{BnACS1}$-Ct$_{BnTIP41}$
0.4
0.2
0
-0.2
-0.4
ΔCt
1
2
3
4
cDNA dilution
y = 0.0207x - 0.1452
R² = 0.3211

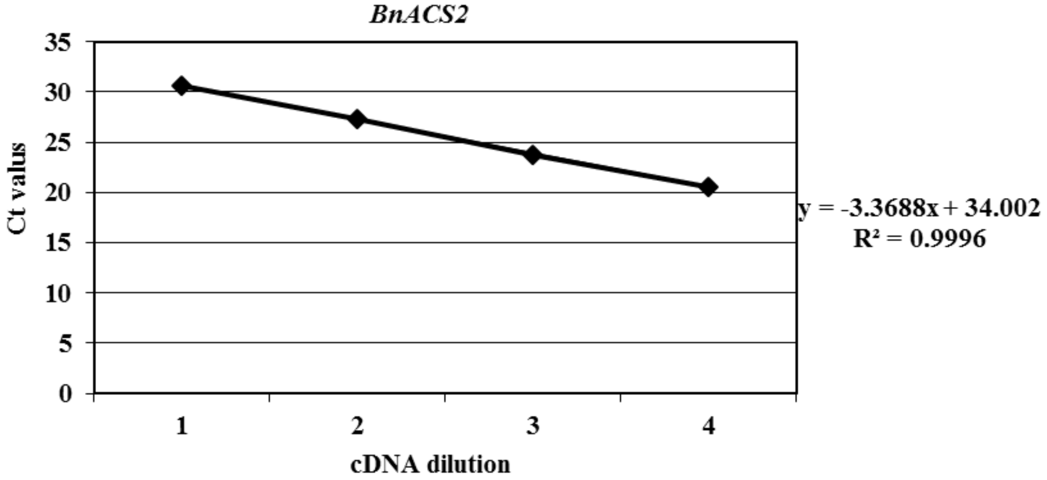
BnACS2
35
30
25
20
15
10
5
0
Ct valus
1
2
3
4
cDNA dilution
y = -3.3688x + 34.002
R² = 0.9996

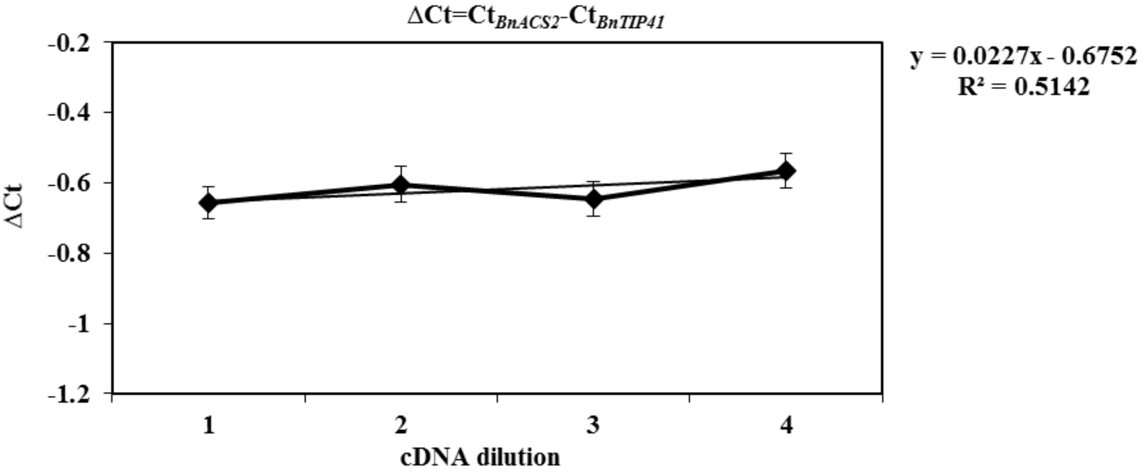
ΔCt=Ct$_{BnACS2}$-Ct$_{BnTIP41}$
ΔCt
-0.2
-0.4
-0.6
-0.8
-1
-1.2
1
2
3
4
cDNA dilution
y = 0.0227x - 0.6752
R² = 0.5142

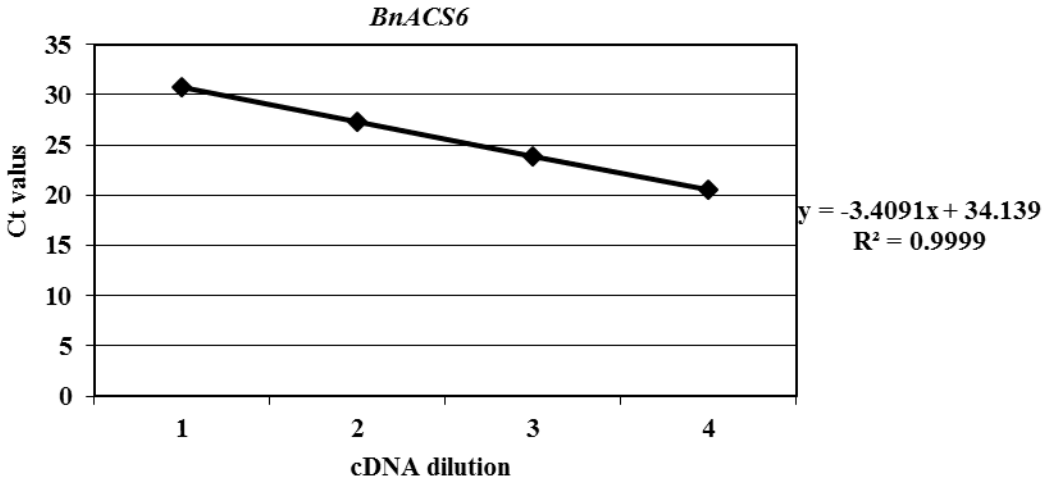
BnACS6
Ct valus
35
30
25
20
15
10
5
0
1
2
3
4
cDNA dilution
y = -3.4091x + 34.139
R² = 0.9999

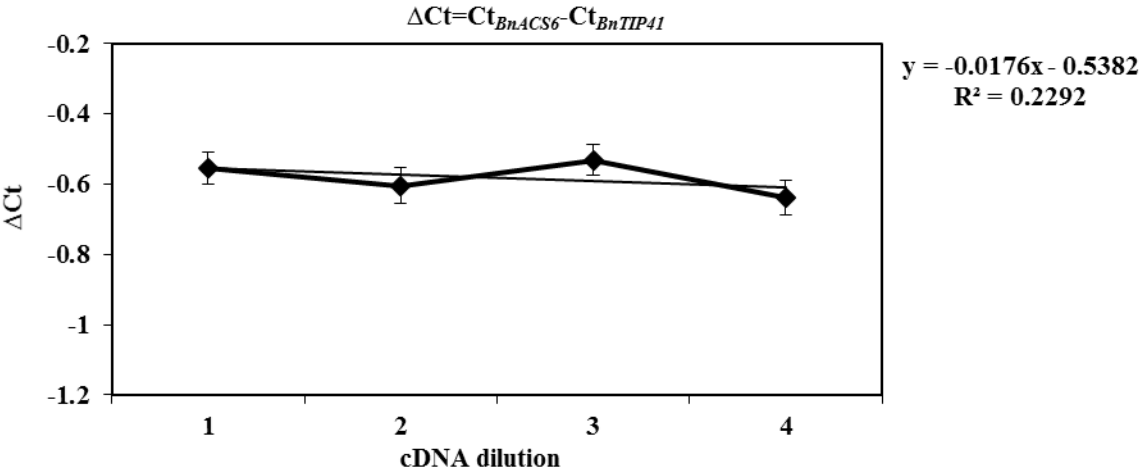
ΔCt=Ct$_{BnACS6}$-Ct$_{BnTIP41}$
ΔCt
-0.2
-0.4
-0.6
-0.8
-1
-1.2
1
2
3
4
cDNA dilution
y = -0.0176x - 0.5382
R² = 0.2292

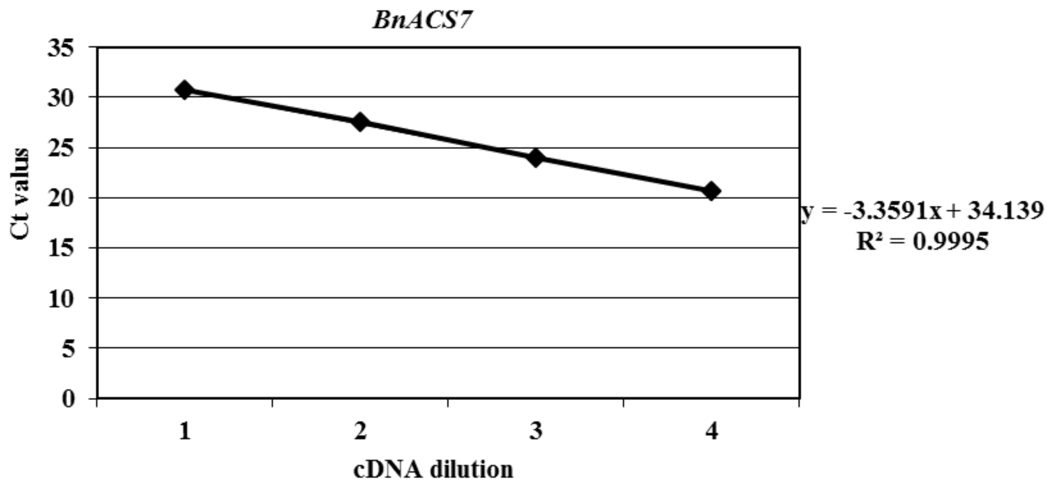
BnACS7
Ct valus
cDNA dilution
y = -3.3591x + 34.139
R² = 0.9995

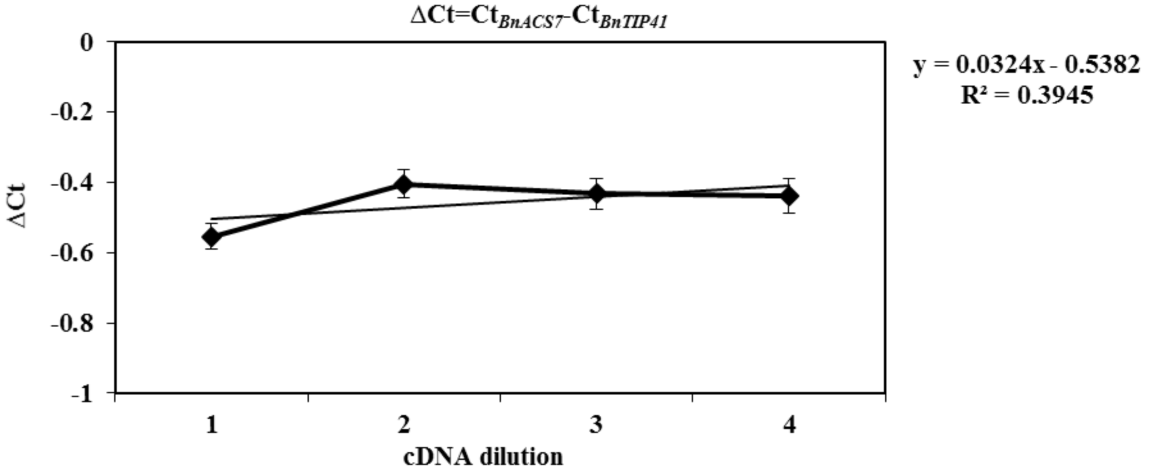
ΔCt=Ct_BnACS7-Ct_BnTIP41
ΔCt
cDNA dilution
y = 0.0324x - 0.5382
R² = 0.3945

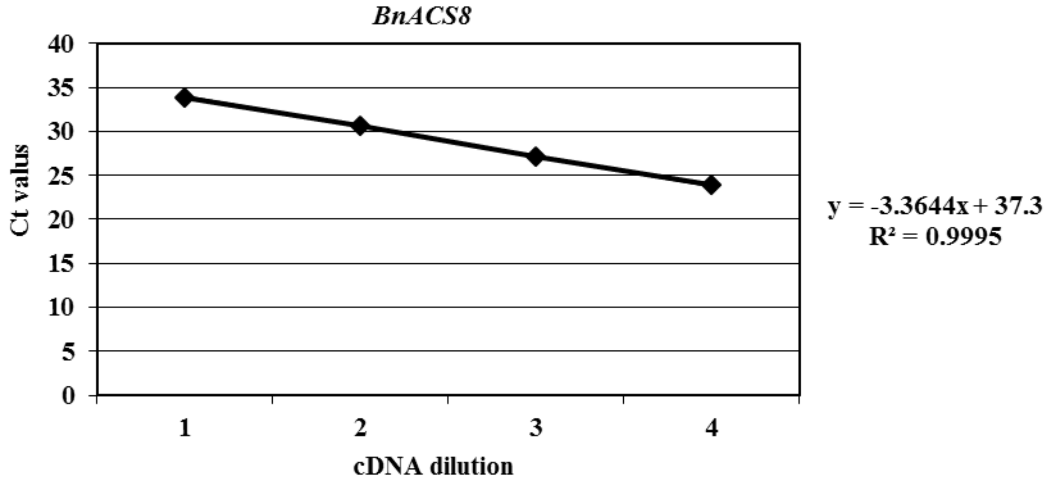
BnACS8
Ct valus
cDNA dilution
y = -3.3644x + 37.3
R² = 0.9995

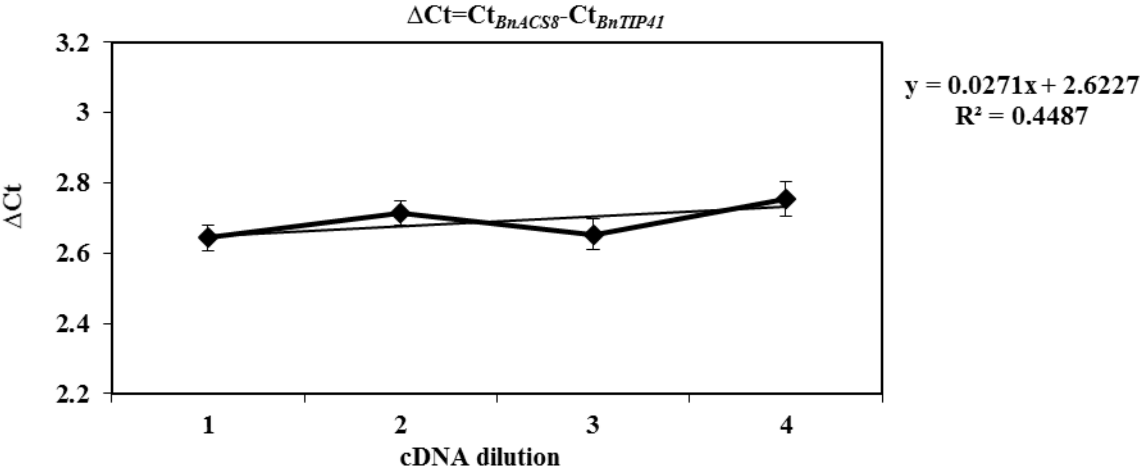

cDNA dilution (1= 0.001; 2= 0.01; 3= 0.1;4=1)
