# Supplementary material for: BnaMPK3 Is a Key Regulator of Defense Responses to the Devastating Plant Pathogen Sclerotinia sclerotiorum in Oilseed Rape
Source: Front Plant Sci. 2019 Feb 8;10:91. doi: 10.3389/fpls.2019.00091 (PMC6376111; doi:10.3389/fpls.2019.00091)
Supplement: FILE S3 — A disease index used to evaluate the symptom severity at maturity. [file Data_Sheet_3.PDF]

Supplementary File 3

A disease index used to evaluate the symptom severity at maturity.

Table 1 Severity scores for stem rot disease caused by *Sclerotinia sclerotiorum* on oilseed rape (*Brassica napus*) at maturity in China (Zhou et al. 1993)[a]

Score	Severity of symptoms
	Percentage stem circumference with lesions (PS) or percentage branches with lesions (PB)
0	No lesion
1	PS<25 or PB<30
2	25⩽PS<50 or 30⩽PB<60
3	50⩽PS<75 or PB⩾60
4	PS⩾75 or almost all branches with lesions

[a]This is the Chinese national protocol for scoring sclerotinia stem rot. All plots are scored by both the PB/PS and the PP methods. Usually the two methods produce the same score. If epidemics are late, PP may produce a lower score than PB/PS and the PP score is used as the final score.

Resistance was evaluated at maturity as a disease index (DI). DI = $\sum(I\, n_I)/(N\, k)$, where I is a disease severity score on the 0 – 4 scale (Zhou et al. 1993, Table 1), n_I is number of plants with each score, N is total number of plants assessed and k is the highest score (here it is 4).

Reference:

Zhou, B. Chen, D. Yu, Q. Liu, S., and Yang, J. (1993). Screening and breeding for multiple resistance to *Sclerotinia* stem rot, downy mildew and virus disease. *Chinese J Oil Crops* 16:14–17.
